# Mechanism of Antiradical Activity of Coumarin-Trihydroxybenzohydrazide Derivatives: A Comprehensive Kinetic DFT Study

**DOI:** 10.3390/antiox13020143

**Published:** 2024-01-24

**Authors:** Žiko Milanović, Dušan Dimić, Edina H. Avdović, Dušica M. Simijonović, Đura Nakarada, Vladimir Jakovljević, Radiša Vojinović, Zoran S. Marković

**Affiliations:** 1 Department of Science, Institute for Information Technologies, University of Kragujevac, Liceja Kneževine Srbije 1A, 34000 Kragujevac, Serbia; ziko.milanovic@uni.kg.ac.rs (Ž.M.); edina.avdovic@pmf.kg.ac.rs (E.H.A.); dusicachem@kg.ac.rs (D.M.S.); zmarkovic@uni.kg.ac.rs (Z.S.M.); 2 Faculty of Physical Chemistry, University of Belgrade, Studentski trg 12-16, 11000 Belgrade, Serbia; ddimic@ffh.bg.ac.rs (D.D.); djura@ffh.bg.ac.rs (Đ.N.); 3 Faculty of Medical Sciences, University of Kragujevac, Svetozara Markovića 69, 34000 Kragujevc, Serbia; drvladakgbg@yahoo.com; 4 Department of Natural Science and Mathematics, State University of Novi Pazar, Vuka Karadžića bb, 36300 Novi Pazar, Serbia

**Keywords:** coumarin, free radicals, antioxidant, gallic acid, QM-ORSA

## Abstract

As part of this study, the mechanisms of the antioxidant activity of previously synthesized coumarin–trihydrobenzohydrazine derivatives were investigated: (*E*)-2,4-dioxo-3-(1-(2-(2″,3″,4″-trihydroxybenzoyl)hydrazineyl)ethylidene)chroman-7-yl acetate (**1**) and (*E*)-2,4-dioxo-3-(1-(2-(3″,4″,5″-trihydroxybenzoyl)hydrazineyl)ethylidene)chroman-7-yl acetate (**2**). The capacity of the compounds to neutralize HO^•^ was assessed by EPR spectroscopy. The standard mechanisms of antioxidant action, Hydrogen Atom Transfer (HAT), Sequential Proton Loss followed by Electron Transfer (SPLET), Single-Electron Transfer followed by Proton Transfer (SET-PT), and Radical Adduct/Coupling Formation (RAF/RCF) were examined using the QM-ORSA methodology. It was estimated that the newly synthesized compounds, under physiological conditions, exhibited antiradical activity via SPLET and RCF mechanisms. Based on the estimated overall rate constants (*k*_overall_), it can be concluded that **2** exhibited a greater antiradical capacity. The obtained values indicated a good correlation with the EPR spectroscopy results. Both compounds exhibit approximately 1.5 times more activity in comparison to the precursor compound used in the synthesis (gallic acid).

## 1. Introduction

The primary source of food degradation, alongside microbiological decomposition, is food oxidation, which results in the development of rancidity [1]. During the 20th century, antioxidants were introduced into the food industry as a significant strategy for reducing the degradation of stored food caused by oxidation. This development has played a crucial role in the advancement of the food industry [2]. The mechanism by which antioxidants reduce the amount of reactive radical species significantly improves their protective efficacy against oxidation, enhances nutritional quality, and serves as a preventive measure against food spoilage [3]. The ingestion of antioxidants in the body leads to the establishment of homeostasis in the production of highly reactive radical species. Specifically, increased activity of various exogenous and endogenous factors inside an organism can result in an imbalance and uncontrolled generation of reactive radical species. This disturbance disrupts the protective mechanisms of the cell and causes oxidative stress [4]. The primary role of antioxidants is to inhibit intracellular oxidation while preserving cellular equilibrium, thereby contributing to the promotion of health and prevention of various diseases, including neurodegenerative [5], cardiovascular [6], gastrointestinal [7], and numerous other medical conditions and diseases [8].

The use of synthetic antioxidants, which are more efficient and cost-effective in food processing and preservation, has been widely adopted. The available literature provides evidence for synthesizing hybrids of natural antioxidants with compounds possessing notable pharmacological properties, aiming to increase antioxidant activity. Because of their potential bioactivities, including their potent antioxidant effects, coumarin derivatives have garnered a great deal of interest among these compounds [9,10]. The available literature on coumarin derivatives suggests that these compounds are effective free radical neutralizers, which are important factors in the development of many chronic diseases, including malignant diseases [11], neurological disorders [12], inflammatory processes (rheumatoid arthritis, vasculitis) [13], cardiovascular disorders [14] and many other conditions known as premature ageing [15]. These properties arise from their ability to interact directly with reactive radical species, interrupt oxidation chain events, and protect biological components from oxidative stress damage. In chemistry and molecular biology, understanding biological processes and developing potential new medications and therapies relies heavily on knowing the principles of the mechanism of antiradical activity [16].

**Figure 1:**
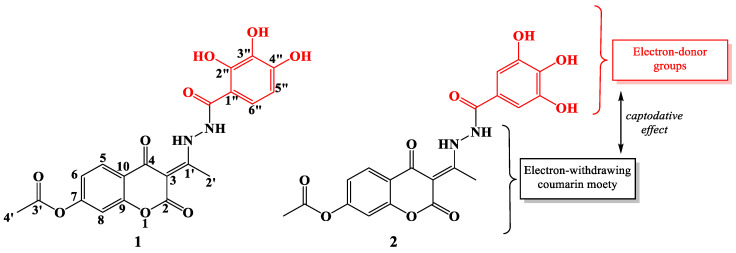
2D structures of investigated compounds **1** (left) and **2** (right) with atomic numbering.

The synthesis of new and potentially better antioxidants has been a challenge for scientists in recent decades. Special emphasis was placed on the synthesis of compounds designed to possess antioxidant properties comparable to those of natural antioxidants. The advantage is that these compounds are often more stable and cheaper to produce, and better stability of food products can be achieved. As part of this study, the mechanisms of antiradical activity of previously synthesized coumarin-hydroxybenzohydrazide derivatives of polyhydroxy acids: (*E*)-2,4-dioxo-3-(1-(2-(2″,3″,4″-trihydroxybenzoyl)hydrazineyl)ethylidene)chroman-7-yl acetate (**1**) and (*E*)-2,4-dioxo-3-(1-(2-(3″,4″,5″-trihydroxybenzoyl)hydrazineyl)ethylidene)chroman-7-yl acetate (**2**) were investigated (Figure 1) [17].

Both compounds, which were synthesized using green chemistry principles, were selected as objects of interest because of their very good activity in the fight against oxidative stress. According to the study’s findings, using standard antioxidant activity assays (DPPH, ABTS, FRAP), the investigated compounds exhibited exceptionally high levels of antioxidant activity [17]. A comprehensive understanding of antioxidant activity necessitates a profound understanding of the mechanisms underlying the direct reactions between the compounds under investigation and highly reactive radical species. For this reason, we investigated the mechanisms of antiradical action between investigation compounds **1**, **2** and HO^•^. The examination was carried out using the Quantum Mechanics-based Test for Overall Free Radical Scavenging Activity (QM-ORSA) methodology [18]. The used methodology involves the determination of thermodynamic and kinetic parameters of several common radical scavenging mechanisms, under physiological conditions, such as Hydrogen Atom Transfer/Proton-Coupled Electron Transfer (HAT/PCET), Single-Electron Transfer followed by Proton Transfer (SET-PT), Sequential Proton Loss followed by Electron Transfer (SPLET), and Radical Adduct/Coupling Formation (RAF/RCF) [19,20]. This approach can provide new insights into the interactions between derivatives and radicals, allowing for a better understanding of their antiradical potential and possible application in the development of new drugs.

## 2. Materials and Methods

### 2.1. Chemicals

The compounds under investigation were synthesized and subsequently subjected to recrystallization using the procedure outlined in the previously described publication [17]. The chemicals H_2_O_2_, FeSO_4_, phosphate buffer, and DMSO were obtained from Sigma-Aldrich (St. Louis, MO, USA) as reagents of analytical grade and were utilized without any modifications [21,22]. The spin-trap DEPMPO was procured from Enzo Life Sciences and subsequently purified using the methodology outlined by Jackson [23].

### 2.2. Electron Paramagnetic Resonance (EPR) Spectroscopy Measurement

Electron Paramagnetic Resonance Spectroscopy (EPR) experiments were conducted at room temperature (293 K) using a Bruker Biospin Elexsys II 540 (9.65 GHz) spectrometer. The experimental setup employed the following operating parameters: a power attenuation of 13 dB, modulation amplitude of 2 mT, modulation frequency of 100 kHz, and sweep time of 120 s. The hydroxyl radicals (HO^•^) were generated via conventional Fenton processes, which involved the utilization of 1 mM H_2_O_2_ and 0.33 mM FeSO_4_. Additionally, 0.1 M DEPMPO was introduced as a spin-trapping agent in 100 mM phosphate-buffered solution at pH 7.4. Spectra were obtained two minutes after the introduction of a suitable iron catalyst. Stock solutions of both compounds were prepared at a concentration of 15 mM using dimethyl sulfoxide (DMSO) as the solvent. The stock solutions were then diluted with water to a final concentration of 5 μM. The solutions were prepared using dimethyl sulfoxide (DMSO) as the solvent because the compounds exhibited minimal solubility in water. Subsequent dilution was conducted to replicate the aquatic environment simulated in the theoretical portion of this study. The blank sample was composed of an equivalent quantity of dimethyl sulfoxide (DMSO) to the samples containing **1** and **2**. Determination of the reactivity of the compounds towards HO^•^ involved measuring the relative decrease in the average intensity of the two most prominent peaks, including both the positive and negative part, of the DEPMPO–HO^•^ adduct in the low-field region of the spectrum. The outcome is shown as the percentage of radical reduction (% RR), which is calculated as follows:

% RR = 100 × (I_0_ − I_a_)/I_0_ (1)

where I_a_ and I_0_ are the intensities of peaks of DEPMPO–HO^•^ adduct with and without **1** and **2**.

### 2.3. Theoretical Calculations

All computations in this study were performed using the *Gaussian09* software package (Revision A.02) [24]. All structures were optimized using the M06-2X/6-311++G(d,p) theoretical model [25] in combination with the CPCM polarizable continuous solvation model without geometrical constraints [26] The M06-2X functional is recommended for the computation of interatomic interactions at short- and medium-range distances in kinetic calculations. It has been effectively employed by various researchers for this purpose [27,28,29,30]. Stationary points (local minima and transition states) were characterized by the number of imaginary frequencies: transition states had one imaginary frequency, whereas equilibrium geometries did not possess any imaginary frequencies.

#### Calculation of the Rate Constants

The mechanisms underlying the antiradical activity of **1** and **2** against the HO^•^ radical were investigated using the QM-ORSA approach [18]. The present methodology involves the determination of the kinetic parameters associated with thermodynamically favorable reaction pathways. Subsequently, the +determination of the overall rate constant (*k*_overall_) served as a quantitative measure of the antiradical efficacy of the compound. The *k*_overall_ values (Equations (S17)–(S19)) were calculated by combining the product of the total rate constant (*k*_TOT_) (Equation (S20)) and estimated values of the molar fraction (*f*) of the acid-base species of the investigated compounds (Equations (S1)–(S16)). The studied compounds are expected to exist in different acid-base forms in aqueous solutions with a pH within the range of physiological conditions. These different forms can interact with free radicals. Therefore, it is important to determine *p*K_a_ values and molar fractions before evaluating their antioxidant capacity. The *p*K_a_ values for both compounds were determined using the ACD/pKa software package based on the following justifications [31]. Additionally, the *k*_TOT_ values were calculated as the sum of the corresponding rate constants of the operative reaction pathways.

The first phase of the QM-ORSA process includes the evaluation of thermodynamic parameters of standard mechanisms of antioxidant action. The values of the Gibbs free energy change Δ_r_*G* were determined at standard conditions, specifically at a temperature of 298.15 K and a pressure of 101,325 Pa. If the reaction is exergonic (Δ_r_*G* < 0) or isogonic (Δ_r_*G* ≤ 0), it can be concluded that the reaction path is thermodynamically favorable. For the thermodynamically favorable reaction pathways, the corresponding kinetic parameters were calculated. All examined reactions are bimolecular [32]. If a transition state geometry exists between the reactant and product (HAT and RAF mechanisms), the determination of the rate constant can be obtained by the use of standard transition state theory (TST) or Eyring’s equation [33]:

(2) $$k_{TST}=\frac{k_{B}T}{h}\exp\left(\frac{-\Delta G_{a}^{\neq }}{RT}\right)$$

where k_B_ and h stand for the Boltzmann and Planck constants, T is temperature, R is the gas constant (8.314 J mol^−1^ K^−1^) and $∆G_{a}^{\neq }$ is the Gibbs free energy of activation.

When examining the HAT and RAF processes, it is imperative to incorporate the degeneracy of the reaction path, *σ*, and the transmission coefficient, γ (T) into the previous equation. Consequently, the Eyring equation transforms, resulting in:

(3) $$k_{ZCT\_0}=\sigma \gamma (T)\frac{k_{B}T}{h}\exp\left(\frac{-\Delta G_{a}^{\neq }}{RT}\right)$$

*TheRate* software was employed to determine rate constants [34]. The rate constants were obtained for the standard state of 1 M using the Eckart method, which is also referred to as ZCT_0 [35]. The energy values and partition functions were obtained by quantum mechanical calculations.

It is well known that transition states cannot be located in processes involving the transition of electrons from one chemical species (i.e., atom, molecule, free radical, ion, etc.) to another. This means that it is not possible to describe the path of electron movement in mechanisms involving electron transfer such as SET-PT and SPLET mechanisms. Marcus’ theory was applied to estimate the reaction barrier, in these particular instances [36].

## 3. Results

### 3.1. EPR Determination of the Reactivity of ***1*** and ***2*** towards HO^•^

**Figure 2:**
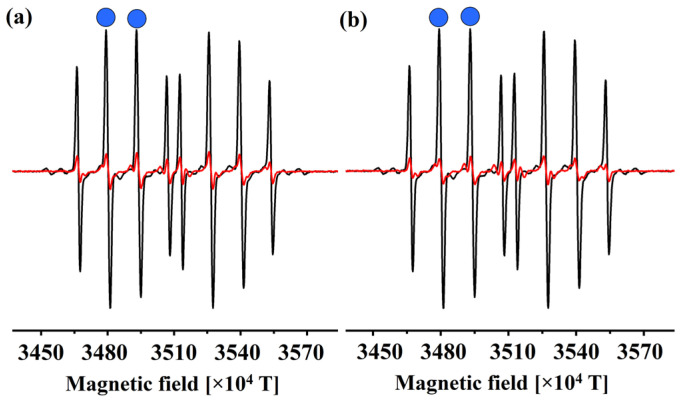
The DEPMPO–HO^•^ adduct solution’s EPR spectra in the presence (red line) and absence (black line) of the compounds (**a**) **1** and (**b**) **2**. The blue dots represent the peaks whose height was measured.

The reactivity of **1** and **2** to HO^•^ was studied using electron paramagnetic resonance (EPR). Radicals were produced within the Fenton system and a characteristic DEPMPO–HO^•^ signal was observed in the spectrum. To simulate the physiological conditions of measurement, the pH of the solution was adjusted to 7.4 with a phosphate-buffered solution. The blank contained the radical generation system, excluding the examined coumarin derivatives (Figure 2).

The spectra were recorded two minutes after the reaction began, as this is sufficient time for all of the compounds to react without spontaneous degradation of the adduct. Upon the addition of compounds **1** and **2** the DEPMPO–HO^•^ signal intensity was reduced, which proves that these compounds exert antiradical scavenging potential. These two compounds reduced the DEPMPO–HO^•^ signal by 87% (**1**) and 90% (**2**). Both compounds have three hydroxyl (‒OH) groups attached to the aromatic ring. Therefore, it is evident that the position of these groups plays a crucial role in the antioxidant activity of these compounds. Furthermore, it is suggested that the examination of acid-base equilibria, which will be discussed in the subsequent section, holds significant relevance in elucidating the observed experimental outcomes. In the final section, the estimated k_overall_ values are compared with the outcomes of the experimental studies.

### 3.2. Thermodynamic Analysis

The determination of acidity or pK_a_ value plays a crucial role in determining the physicochemical properties of compounds, including hydrophobicity, lipophilicity, and polarizability. Determination of the molar fraction of acid-base species in compounds allows for a more comprehensive exploration of the mechanisms of antioxidant activity. Specifically, under these specified conditions, the compounds studied, **1** and **2**, can exist in several acid-base forms. Deprotonation routes and the molar fraction of acid-base species were determined based on the observed pK_a_ values of the compounds (Figures S1 and S2) and subsequent calculations (Equations (S1)–(S16)). 

Based on the estimated molar fractions, it was observed that in an aqueous solution at physiological pH, the most abundant acid-base species of compound **1** are **H_4_A** (60.3%), **H_3_A^−^** (30.9%), and **H_2_A^2−^** (8.5%). In addition, the dominant acid-base species for compound **2** are **H_4_A** (79.3%) and **H_3_A^−^** (19.9%). The evaluation of the antioxidant activity mechanisms of polyhydroxy compounds at a specific pH value has been the subject of earlier research, primarily because of the presence of many acid-base forms of the compounds being studied [27,28,29,30]. Standard mechanisms of antioxidant action: HAT/PCET, SET-PT, SPLET, and RAF, involving neutral species **H_4_A** and the HO^•^ radical, are presented in the Supplementary Material (Equations (S23)–(S28)). On the other hand, the reaction pathways for monoanionic species **H_3_A^−^** are represented by the following equations:

**SET**: H_3_A^−^ + HO^•^ → H_3_A^•^ + HO^−^ (4)

**PT**: H_3_A^•^ + HO^−^ → H_2_A**^•^**^−^ + H_2_O (5)

**HAT/PCET**: H_3_A^•^ + HO^•^ → H_2_A (hin) + H_2_O (6)

**RCF**: H_3_A^•^ + HO^•^ → [H_3_A–OH] (7)

To interpret the mechanisms of antiradical activity of compound **2**, it is important to consider the presence of a dianionic species, namely **H_2_A^2−^** (Equations (8)–(11)). The mechanisms associated with this species are represented by the following equations:

**SET**: H_2_A^2−^ + HO^•^ → H_2_A**^•^**^−^ + HO^−^ (8)

**PT**: H_2_A**^•^**^−^ + HO^−^ → HA^2−^**^•^** + H_2_O (9)

**HAT/PCET**: H_2_A**^•^**^−^ + HO^•^ → HA^−^ (hin) + H_2_O (10)

**RCF**: H_2_A**^•^**^−^ + HO^•^ → [H_2_A–OH]^−^ (11)

**Figure 3:**
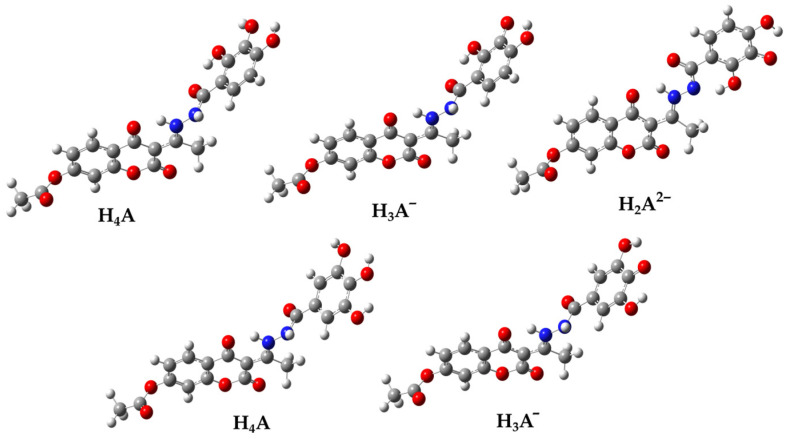
The optimized geometries of the acid-base species **1** (up) and **2** (down) in an aqueous solution determined by using the M06-2X/6-311++G(d,p) level of theory, in combination with the CPCM solvation model. Legend: grey—carbon atom, white—hydrogen atom, red—oxygen atom, blue—nitrogen atom.

By analyzing the geometries of the acid-base species, it was expected that the structure **H_2_A^2−^** (Figure 3) would be stabilized by the intramolecular hydrogen bond O2″–H---N2. Despite numerous attempts, the equilibrium geometry of this structure has not been found. Specifically, in the optimization process, the hydrogen atom originating from the –NH group undergoes a spontaneous transition to the O-2″ atom, resulting in the formation of a significantly more stable planar geometry. For this reason, further studies on the mechanism of antiradical activity were performed on the more stable acid-base **H_2_A^2−^** species (Figure 3).

**Scheme 1:**
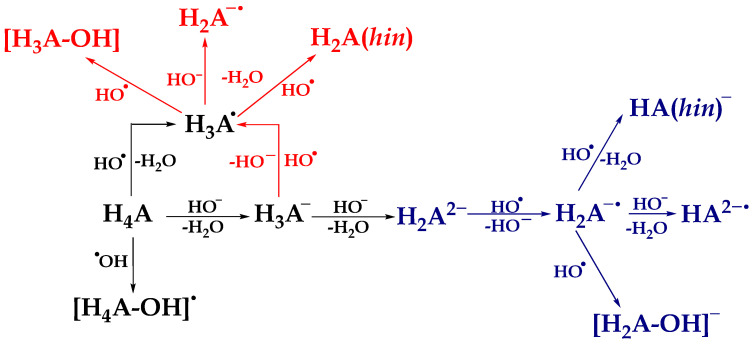
Thermodynamically favored reaction pathways between the most abundant acid-base species of investigated compounds and reactive radical species HO^•^. The black color describes the thermodynamically favored reaction mechanisms between neutral species **H_4_A** and HO^•^. The red color describes the favored reaction mechanisms between monoanionic species **H_3_A^−^** and HO^•^, while the blue color describes the reaction mechanisms between dianionic species **H_2_A^2−^** and HO^•^.

The scheme of thermodynamically favored reaction paths is presented in Scheme 1, while the values of reaction free energies (Δ_r_G, kJ mol^−1^) of standard mechanisms of antioxidative action are found in Table 1. In addition, a two-dimensional representation of the geometry of the products that are formed between the investigated acid-base species and HO^•^ are presented in Tables S1 and S2.

First, estimated values of the thermodynamic parameters for the neutral species **H_4_A** were analyzed. The investigated compounds have a total of four positions, namely three hydroxyl groups (–OH) and one amino group (–NH), from which the transfer of hydrogen atoms (HAT/PCET) to HO^•^ is possible. The distinctly exergonic Δ_r_G_HAT/PCET_ values ranging from −134 to −165 kJ mol^−1^ show that the HAT/PCET mechanism is operational at all positions of both investigated compounds (Table 1). As expected, the transfer of the hydrogen atom from the 2″-OH (−157 kJ mol^−1^, **1**) and 4″-OH (−165 kJ mol^−1^, **2**) positions is thermodynamically favored. This conclusion is supported by the stabilization of the radical species by intramolecular hydrogen bonding, as shown in Figure S3. The enhanced stability of the **H_3_A^•^** radical at position 4″ of compound **2** can be attributed to the improved delocalization of the unpaired electron through the ortho (C3″ and C5″) and para (C1″) atoms, as well as the C=O and N2-H groups. Compounds **1** and **2** exhibited significant exergonic Δ_r_G_HAT/PCET_ values for the hydrogen atom transfer from the N2–H groups. This can be explained by the stabilization of the newly formed planar radical structures as well as the undisturbed delocalization of the unpaired electron along the hydroxybenzohydrazine and coumarin moieties (Figure S3). The higher reactivity of radical at the 2-N^•^ location of **1** can be explained by better delocalization of the unpaired electron via the C2″, C6″ and C4″ atoms of the hydroxybenzohydrazine moiety. The dihedral angles N2–C5–C1–C2″ and N2–C5–C1–C6″ exhibit differences in values, specifically 179.6° and 0.3° for **1** (2-N^•^) and 178.9° and 1.3° for **2** (2-N^•^). These differences can be attributed to the presence of a hydrogen bond, O2″–H---O5′, in **1** (2-N^•^), which plays a role in stabilizing the system and maintaining its planarity (Figure S3). This difference is a consequence of a better distribution of the unpaired electron which may explain these differences and the lower reactivity of **1** (2-N^•^) compared to **2** (2-N^•^).

There exists a total of fourteen potential binding sites of highly reactive radical species for **1** and **2** whereby they lead to a formation of radical adducts (RAF mechanism). Figures S4 and S5 illustrate the optimized geometries of the radical adducts, while Table 1 summarizes of the Δ_r_G_RAF_ values. The estimated range of the Δ_r_G_RAF_ values was −59 to 1 kJ mol^−1^ for **1** and −63 to 3 kJ mol^−1^ for **2**. The electrophilic nature of HO^•^ indicates a preference for attacking the C1′ and C6″ positions in both compounds. Examination of the formed adducts showed that the carbon atom to which the –OH radical was attached underwent rehybridization from sp^2^ to sp^3^. This rehybridization disrupted the planarity and aromaticity of the system. However, it is noteworthy that the overall geometry of the adducts was additionally stabilized by the formation of intramolecular hydrogen bonds with the neighboring coumarin carbonyl group and the amino group of hydrazine moiety.

Similar to the HAT/PCET process, the spontaneity of the proton loss (SPL) by HO– was observed at all four potential positions. This can be seen from the exergonic Δ_r_G_SPL_ values, which range from −128 to −168 kJ mol^−1^ for **1** and from −123 to −146 kJ mol^−1^ for **2** (Table 1). The proton transfer from the N2-H groups of **H_4_A** of both compounds is the most thermodynamically favorable, as evidenced by the significantly exergonic Δ_r_G_SPL_ values of −168 kJ mol^−1^ and −146 kJ mol^−1^, respectively. This is in agreement with the distribution of the highest occupied molecular orbital (HOMO) of the resulting anion products, as shown in Figure S6. Upon loss of the proton, the anionic products adopt a planar geometry, resulting in a highly homogenous distribution of electron density across both the coumarin base and hydroxybenzohydrazine ring. Electron transfer (ET), which is the second step of the SPLET mechanism, is thermodynamically favored from the formed phenoxide anions (Table 1). It should be noted that the electron transfer from the 2″-O^−^ (4 kJ mol^−1^) anion of the **1** compound exhibits somewhat endergonic values.

Endergonic Δ_r_G_SET_ values for the first step of the SET-PT mechanism of **1** (127 kJ mol^−1^) and **2** (137 kJ mol^−1^) compounds make this mechanism thermodynamically unfavorable (Table 1). Therefore, the SET-PT mechanism will not be the subject of future kinetic studies.

#### Thermodynamic Investigation of the Reaction between Anionic Species and HO^•^

**Figure 4:**
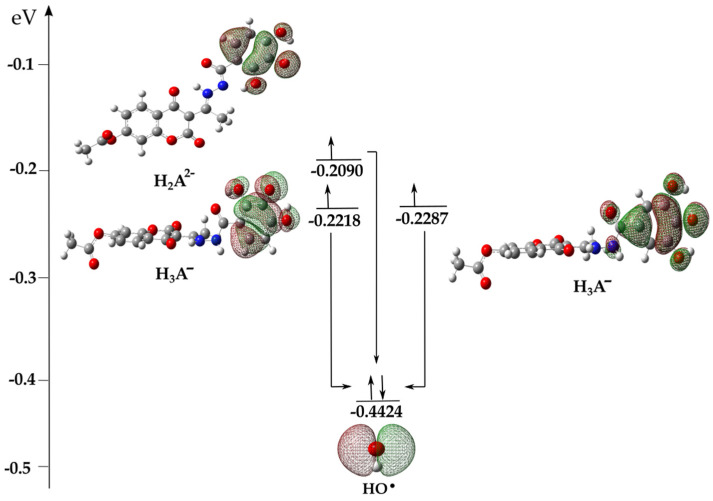
Illustrative representation of the electron transition from the HOMO orbitals of **1** (left) and **2** (right) to the SOMO (Singly Occupied Molecular Orbital) orbital of the HO^•^ with the corresponding values of the orbital energies. Legend: grey—carbon atom, white—hydrogen atom, red—oxygen atom, blue—nitrogen atom.

The complex behavior of the acid-base species present in aqueous solutions is a subject of special discussion. It has already been reported that electron transfer is a logical first step in investigating the mechanisms of the antioxidant properties of acid-base species [28,37,38]. In the presence of HO^•^, an electron from the higher-energy HOMO orbital of the acid-base species **H_3_A^−^** and **H_2_A^2−^** spontaneously, at the rate of diffusion, moves to the lower-energy SOMO orbital of the reactive radical species (Figure 4), thus forming radical products **H_3_A^•^** (−41 kJ mol^−1^), **H_2_A^•−^** (−87 kJ mol^−1^) of compound **1** and **H_3_A^•^** (−23 kJ mol^−1^) of compound **2** (Table 1). The spontaneous transformation of anionic species in the presence of radicals into radical products results in the existence of the above-mentioned species, **H_3_A^•^** and **H_2_A^•−^**, in the aqueous solution. Through further interpretation, it is necessary to investigate the mechanisms of the antiradical action of the formed radical products.

Proton transfer (PT) from **H_3_A^•^** and **H_2_A^•−^** to HO^−^, resulting in the formation of **H_2_A^•−^** and **HA^2−•^** and H_2_O, is an exergonic process (Table 1). The Δ_r_G_PT_ parameters indicate that proton transfer in the 2″-OH (−187 kJ mol^−1^) position of **H_3_A^•^** of compound **1** is thermodynamically most favorable because it is stabilized by an intramolecular hydrogen bond, involving an oxygen atoms electron pair and a proton of the –NH group (Figure S7). The estimated parameters show that proton transfer from the N2-H group of the radical species **H_3_A^•^** (−183 kJ mol^−1^) and **H_2_A^•−^** (−140 kJ mol^−1^) of compound **1**, as well as **H_3_A^•^** (−165 kJ mol^−1^) of compound **2**, is thermodynamically competitive with the previously discussed position. These positions are thermodynamically favorable because of the stabilization of predominantly planar species by the distribution of negative charge along the coumarin base and hydroxybenzohydrazine ring, as well as stabilization by strong intramolecular hydrogen bonds (Figure S7).

Analogous to proton transfer, hydrogen atom transfer (HAT/PCET) from **H_3_A^•^** and **H_2_A^•−^** species with the formation of **H_2_A** and **HA^−^** products are spontaneous in all positions. The hydrogen atom transfer process is thermodynamically most favorable at the 2-OH position of **H_3_A^•^** (−202 kJ mol^−1^) and the 4″-OH position of the **H_2_A^•−^** (−214 kJ mol^−1^) species of **1**, as indicated by their highly strongly exergonic Δ_r_G_HAT/PCET_ values. The high exergonic values observed can be attributed to the stability of the resulting species through the formation of hydrogen bonds between phenoxide oxygen 2″-O and the –NH group proton. On the other hand, the exergonic values for hydrogen atom transfer from the N2-H groups **H_3_A^•^** and **H_2_A^•−^** were found to be lower but still significant. This can be attributed to the planarity of the resulting products and their stabilization through hydrogen bonding, as shown in Figure S8.

The coupling of radical species (RCF) with the formation of neutral ([**H_3_A–OH**]) and monoanionic ([**H_2_A–OH**]^−^) adducts (Figures S9–S11) was the most spontaneous at positions C1″–C6″ of the hydroxybenzohydrazine aromatic ring (Table 1). As expected, significantly higher exergonic values result from the pronounced electrophilicity of HO^•^, which has a greater affinity for positions where the unpaired electron is delocalized (Figure S3).

### 3.3. Kinetic Analysis

Kinetic investigations have been conducted on thermodynamically favorable mechanisms. The rate constants were determined using the TST and ZCT_0 approaches in addition to the Marcus theory. Table 2 presents the estimated reaction rate constants obtained using the ZCT approach, whereas the Supplementary Material contains the values of the rate constants estimated using the TST method (Table S3). The k^ET^ values in Table 2 represent the rate constants calculated using Marcus’s theory.

Hydrogen atom transfer (HAT/PCET) from the –OH groups of neutral **H_4_A** species of both compounds occurs via the transition state geometries shown in Figure S12. The similarity between the values of the rate constants estimated by the TST (Table S3) and ZCT_0 (Table 2) methods suggests that both approaches can be used to estimate rate constants at room temperature. Based on the obtained data, it can be concluded that hydrogen atom transfer exhibits the highest rate constant in the 2″-OH (**1**, 2.22 × 10^7^ M^−1^s^−1^) and 4″-OH (**2**, 3.05 × 10^5^ M^−1^s^−1^) positions. This implies that thermodynamically favored products are also kinetically favorable.

**Figure 5:**
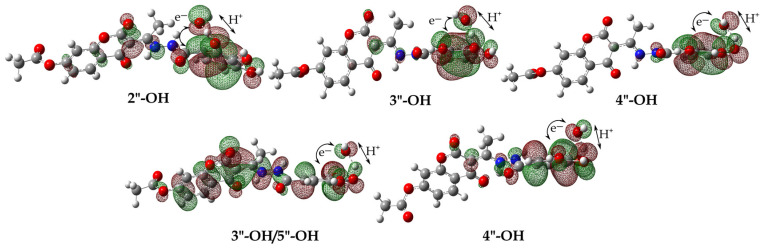
The shape of the SOMO orbitals of the optimized transition state geometries for HAT/PCET reaction pathways between **H_4_A** of **1** (top) and **2** (bottom) compounds and HO^•^ with characteristic intraatomic distances (Å). Legend: grey—carbon atom, white—hydrogen atom, red—oxygen atom, blue—nitrogen atom.

It has been emphasized that hydrogen atom transfer can occur via two reaction pathways: HAT or PCET. Examination of the SOMO orbitals of the transition states offers a more comprehensive understanding of the hydrogen atom transfer mechanism (Figure 5). It can be seen that the SOMO orbitals in the transition states are not localized along the transition vector **HO···H–H_3_A**, but include the proton acceptor *p* orbitals. Examination of the SOMO orbitals reveals a distinct divergence in the pathways of proton and electron transfer, indicating the prevalence of a PCET mechanism across all positions. Specifically, proton transfer occurs from the hydroxyl (–OH) group at different positions, while electron movement takes place from the delocalized electron density of the aromatic ring to the hydroxyl radical (HO^•^).

The geometries of the transition states for the transfer of the hydrogen atoms from the N2–H group were not determined. Therefore, the dependence of the total energy (a.u) on the HO‒HN (Å) distance was monitored as a scanning coordinate, and the results are presented in Figure S13. Based on the displayed coordinates, it can be concluded that the observed reactions occur in the absence of an energy barrier, indicating that their rate constants are controlled by diffusion with a value of 1.91 × 10^9^ M^−1^s^−1^.

The equilibrium geometries of the transition state through which radical adducts are formed (RAF mechanism) are shown in Figures S14 and S15, and the kinetic parameter values are summarized in Table 2 and Table S3. The kinetically most favorable positions for HO^•^ attack on **H_4_A** are: C-3″(1.67 × 10^8^ M^−1^s^−1^) and C-6″(7.29 × 10^7^ M^−1^s^−1^) of **1** compound as well as C-2″ (5.69 × 10^8^ M^−1^s^−1^), C-4″ (7.41 × 10^7^ M^−1^s^−1^) and C-6″ (1.39 × 10^7^ M^−1^s^−1^) of compounds **2** with Δ*G*_a_ values < −30 kJ mol^−1^. A significant difference in the estimated values of the rate constants, as determined by the TST and ZCT_0 methods, can be observed upon the analysis of the indicated positions. The large intermolecular distances in the transition states for the mentioned positions: C-3″ (2.263 Å), C-6″ (2.196 Å) of compound **1** (Figure S14) as well as C-2″ (2.147 Å), C-4″ (2.150 Å) and C-6″ (2.152 Å) of **2** compounds (Figure S15) are accompanied by low Δ*G*_a_ values and large rate constant values (Table 2 and Table S3). This observation confirmed that these transition states corresponded to the geometry of the early transition states. The dependence of ln*k*_TST_, ln*k*_ZCT_0_, as a function of the reciprocal of the temperature was monitored for these positions. The results are shown in Figure S16. Based on the presented results, it can be concluded that the rate constants exhibited significant differences across all temperatures. The differences in the *k*_TST_ and *k*_ZCT_0_ values can be attributed to the tunnelling effect, which decreases rapidly with increasing temperature. In this case, the conventional TST method tends to overstate the values of the rate constants and is unsuitable for accurately determining the rate constants at room temperature.

Despite numerous attempts, the geometry of the transition states in the first step of the SPLET mechanism has not been determined. For this reason, the dependence of the total energy (a.u.) on the characteristic distance HO‒H*_n_* (Å) was monitored as a scanning coordinate, and the results for the selected positions are shown in Figure S17. Based on the displayed coordinates, it can be concluded that these reactions occur without an energy barrier; that is, their rate constants are diffusion-controlled (1.91 × 10^9^ M^−1^s^−1^). On the other hand, the values of the rate constants for the second step of the SPLET mechanism, estimated by Marcus’s theory, are on the order of 10^9^, which indicates that these are very fast reaction constants that are diffusion-controlled (Table 2).

#### Kinetic Investigation of the Reaction between Anionic Species and HO^•^

The species that dominate the reaction media are **H_3_A^•^** and **H_2_A^•−^**, because of diffusion-controlled electron transfer from the acid-base species **H_3_A ^−^** and **H_2_A^2−^** to HO^•^. It is reasonable to assume that further reaction mechanisms (HAT/PCET and RCF mechanisms) between the dominant species **H_3_A^•^** and **H_2_A^•−^** and **HO^•^** radicals take place on two potential energy surfaces, that is, in two spin states. Following the methodology introduced in previous research [28,37,38], the dependence of the energy of the system on the distance between two different spin states was examined.

**Figure 6:**
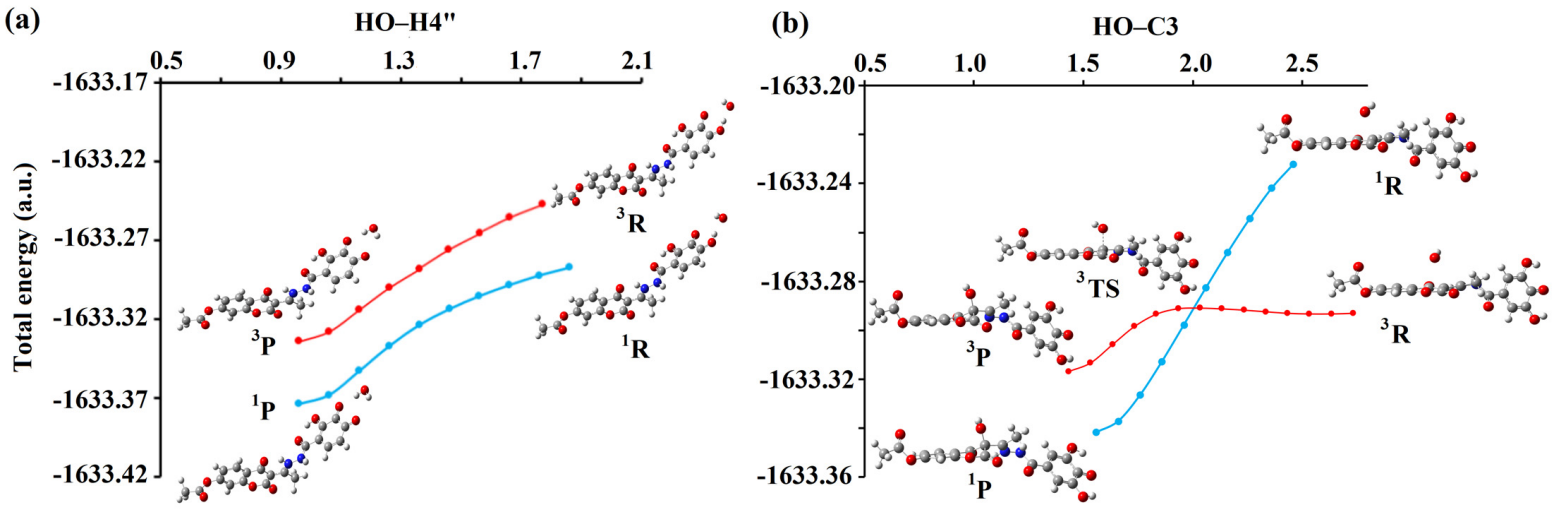
Energy profile for the (**a**) HAT/PCET reaction pathway between **H_3_A^•^** of compound **1** (4″OH) and HO^•^; (**b**) RCF reaction pathway between **H_3_A^•^** of compound **2** (C-3) and HO^•^ in the singlet (blue) and triplet (red) spin states. Interatomic distances are given in angstroms [Å]. Legend: grey—carbon atom, white—hydrogen atom, red—oxygen atom, blue—nitrogen atom.

The results of this study indicate that the hydrogen atom transfer from **H_3_A^•^** and **H_2_A^•−^** to HO^•^ occurs solely in the singlet spin state (blue line), without the involvement of a transition state geometry (Figure 6a and Figure S18). Consequently, this process leads to the formation of neutral singlet products (**^1^P**, blue line), which exhibit greater stability than triplet products (**^3^P**, red line). This implies that the HAT/PCET mechanisms occur in a singlet spin state with a rate constant equivalent to the diffusion rate of 1.91 × 10^9^ M^−1^s^−1^ (Figure 6a and Figure S18).

Further investigations of the addition of HO^•^ with **H_3_A^•^** and **H_2_A^•−^** indicate an identical trend according to which these reactions take place at the rate of diffusion, forming neutral anionic adducts. However, the spin multiplicity changes along the reaction pathway during the interaction involving the C-3 position of **H_3_A^•^** (compound **1**) and HO^•^, as shown in Figure 6b and Figure S19. Specifically, at a significant distance, both species exist together as radical species (**^3^R**), whereas the resulting reaction products are in a stable singlet state (**^1^P**). It was observed that in these reactions, there is a change in spin, that is, reactions occur on two potential energy surfaces. The transformation of reactants to products in the triplet spin state occurs via transition state geometry (**^3^TS**). In contrast, in the singlet spin state, the transformation of **^1^R** to **^1^P** does not involve transition-state geometry. This process is spontaneous, implying that the energy continuously decreases from the reactants to the products. It should be noted that, in all cases, the reactants in the triplet spin state are more stable than the singlet, while the reaction products are far more stable in the singlet spin state. As the two radical species approach each other, the energy of the reaction complex in the triplet state increases slightly, whereas the energy of the reaction complex in the singlet state decreases sharply. The energy change occurs up to the point previously characterized as the Spin Crossing Point, SCP, where spin inversion occurs. At this point, the structures of the complexes in the triplet and singlet spin states were almost identical.

For the reaction between **H_3_A^•^** of compound **1** and HO^•^ the SCP value is at 1.91 Å, while in the reaction **H_3_A^•^** of compound **2** and HO^•^ this value is at 1.99 Å. It is noticeable that there are large differences in the energies of the participants in the reaction between the triplet and singlet states, as expected. The energy difference between the optimized triplet (^3^P) and singlet (^1^P) product in the case of compound **1** is 31 kJ mol^−1^, while in the case of compound **2,** this difference is slightly higher and amounts to 42 kJ mol^−1^. In summary, instead of the reaction going through a demanding and energetically unfavorable transition state (^3^TS), the participants in the reaction go into a far more stable singlet product. This means that the processes described above (RCF mechanism) occur at a rate equal to the rate of diffusion (1.91 × 10^9^ M^−1^s^−1^).

### 3.4. Defining the Plausible Mechanisms of Antioxidant Activity

After evaluating the rate constants of the operative mechanisms of antioxidant activity, the subsequent step involved the estimation of the overall rate constant (k_overall_) under physiological conditions. It has already been said that this value is calculated as the sum of the product of the molar fractions (f) of the corresponding acid-base species and the total rate constant (k_TOT_). Under physiological conditions, the overall rate constants for **1** (3.75 × 10^10^ M^−1^s^−1^) and **2** (4.11 × 10^10^ M^−1^s^−1^) indicate a significant capability for the elimination of HO^•^. The k_overall_ values indicate that **2** exhibits a 1.10-fold greater HO^•^ scavenging capacity than **1** (Table S4). The obtained value is in agreement with the previously discussed EPR spectroscopy results. This comparison further demonstrated the suitability of the QM-ORSA methodology for predicting the antiradical capacity of both existing and newly synthesized compounds.

It has already been emphasized that the ratio between the k_overall_ values of the investigated compounds and the reference compounds is known as the relative antiradical capacity (r^T^, Equation (S21)). This parameter signifies a higher or lower reactivity relative to that of the reference compound. Specifically, the k_overall_ value for Trolox (**Tx**) as a standard antioxidant, estimated under the same reaction conditions, is 1.94 × 10^10^ M^−1^s^−1^ [37]. The obtained r^T^ values indicated a significant level of reactivity exhibited by the investigated compounds in terms of their capacity to inactivate HO^•^ radicals when compared to **Tx** (Table S4). Available literature data indicate that a precursor of investigated compounds, gallic acid (**GA**), has a k_overall_ value of 2.56 × 10^10^ M^−1^s^−1^ [39]. This implied that the investigated compounds were approximately 1.5 times more effective than gallic acid. Therefore, it can be concluded that the presence of a coumarin-based component increases the antioxidant capacity of the newly synthesized compounds.

To determine the involvement of the individual reaction pathways, the relative amount of products, Γ_i_ (%), was estimated (Equation (S22), Table S4). Analysis of Γ_i_ suggests that the HAT/PCET mechanism makes a minimal contribution to the overall antioxidant activity of both compounds. This contribution primarily occurs through the reaction of acid-base species. The RAF mechanism makes a minimal contribution, whereas the RCF mechanism plays a more substantial role in the formation of the product. On the other hand, anions were produced by an equal distribution, resulting in a cumulative proportion of 12.3% for **1** and 14.7% for **2** for all positions. Radicals arising from electron transfer were collectively represented by 37.8% for **1** and 57.6% for **2**. These results demonstrate that the SPLET and RCF mechanisms play a predominant role in the inactivation of HO^•^.

## 4. Conclusions

This study aimed to comprehensively investigate the antiradical activity of coumarin-hydrobenzohydrazine derivatives of hydroxybenzoic acid **1** and **2**. The investigation was performed using Electron Paramagnetic Resonance (EPR) spectroscopy in conjunction with sophisticated computational methods. The theoretical analysis of the reaction between the selected compounds and the HO^•^ used various reaction pathways, namely, Hydrogen Atom Transfer/Proton-Coupled Electron Transfer (HAT/PCET), Radical Adduct Formation (RAF), Single Electron Transfer (SET), Proton Transfer (PT), Sequential Proton Loss (SPL), and Electron Transfer (ET). The EPR spectra exhibit a notable decrease in the abundance of HO^•^ when **1** (87%) and **2** (90%) are present. Based on a qualitative theoretical examination of the results obtained, it is evident that both compounds, under physiological conditions, primarily exhibit antiradical action through the SPLET and RCF pathways. Based on quantitative theoretical analysis, the **2** compound exhibits approximately 1.10 times more activity than the **1** compound. However, when compared to the commercially available antioxidant Trolox (**Tx**), they demonstrated roughly 20 times more activity. In addition, it is essential to note that the antioxidant activity of both compounds was approximately 1.5 times greater than gallic acid (**GA**), one of the starting compounds in these syntheses, which unambiguously means that the coumarin moiety improved the antioxidant capacity of the obtained compounds.

## Figures and Tables

**Table 1:** Estimated values of reaction free energies (Δ_r_G, kJ mol^−1^) of standard reaction mechanisms of antioxidant action between the investigated compounds and HO^•^.

Compounds	Acid-Base Species	Position	HO^•^				
			HAT/PCET	SET-PT		SPLET	
			Δ_r_*G*_HAT/PCET_	Δ_r_*G*_SET_	Δ_r_*G*_PT_	Δ_r_*G*_SPL_	Δ_r_*G*_ET_
**1**	**H_4_A**	**2″-OH**	−152	127	−280	−156	4
		**3″-OH**	−157		−284	−116	−41
		**4″-OH**	−134		−262	−128	−6
		**2-NH**	−142		−269	−168	26
	**H_3_A** **^−^**	**2″-OH**	−202	−41	−187	/	/
		**4″-OH**	−194		−156		
		**2-NH**	−182		−183		
	**H_2_A** **^2−^**	**4″-OH**	−214	−87	−94	/	/
		**2-NH/2″-OH**	−158		−140		
**2**	**H_4_A**	**3″-OH/5″-OH**	−145	133	−278	−125	−20
		**4″-OH**	−165		−299	−142	−23
		**N-H**	−136		−270	−146	9
	**H_3_A** **^−^**	**3″-OH**	−184	−23	−161	/	/
		**5″-OH**					
		**2-NH**	−98		−165		
**Position**			**RAF/RCF**				
			**1**			**2**	
			**H_4_A**	**H_3_A** **^−^**	**H_2_A** **^2−^**	**H_4_A**	**H_3_A** **^−^**
**C-3**			−11	−46	−75	−12	−69
**C-5**			−40	−124	5	−44	−143
**C-6**			−20	5	34	−28	−6
**C-7**			−43	102	−20	−42	75
**C-8**			−27	9	40	−35	−14
**C-9**			−37	120	1	−36	91
**C-10**			1	46	76	3	21
**C-1′**			−59	−63	−107	−60	5
**C-1″**			1	−175	−124	−8	−172
**C-2″**			−50	−187	−161	−42	−108
**C-3″**			−38	−194	−120	−54	−167
**C-4″**			−57	−169	−163	−49	−70
**C-5″**			−42	−84	−83	−26	−170
**C-6″**			−54	−195	−183	−63	−107

**Table 2:** Estimated values of kinetic parameters: activation energy (ΔG_a_, kJ mol^−1^), rate constants of the bimolecular chemical reaction (M^−1^s^−1^) between the investigated compounds and HO^•^ estimated by the Eckart method (k_ZCT_0_) and by Marcus theory (k^ET^).

Compounds	Acid-Base Species	Position	HAT/PCET			SPLET			
			$∆G_{a}^{HAT}$	$k_{ZCT\_0}^{HAT}$		$∆G_{a}^{SPL}$	$k^{SPL}$	$∆G_{a}^{ET}$	$k^{ET}$
**1**	**H_4_A**	**2″-OH**	58	1.94 × 10^4^		~0	1.91 × 10^9^	1	8.02 × 10^9^
		**3″-OH**	41	2.22 × 10^7^				10	7.57 × 10^9^
		**4″-OH**	55	9.22 × 10^4^				2	8.01 × 10^9^
		**2-NH**	~0	1.91 × 10^9^				26	1.47 × 10^8^
**2**	**H_4_A**	**3″-OH/** **5″-OH**	59	3.15 × 10^4^		~0	1.91 × 10^9^	0	8.02 × 10^9^
		**4″-OH**	54	3.05 × 10^5^				0	8.02 × 10^9^
		**2-NH**	~0	1.91 × 10^9^				14	5.84 × 10^9^
**Compounds**	**Acid-Base Species**	**Position**	**HAT/PCET**			**SET-PT**			
			$∆G_{a}^{HAT}$	$k_{ZCT\_0}^{HAT}$		$∆G_{a}^{SET}$	$k^{SET}$	$∆G_{a}^{PT}$	$k^{PT}$
**1**	**H_3_A** **^−^**	**2″-OH**	~0	1.91 × 10^9^		10	7.57 × 10^9^	~0	1.91 × 10^9^
		**4″-OH**							
		**2-NH**							
	**H_2_A** **^2−^**	**4″-OH**	~0	1.91 × 10^9^		35	4.89 × 10^6^	~0	1.91 × 10^9^
		**2-NH**							
**2**	**H_3_A** **^−^**	**3″-OH**	~0	1.91 × 10^9^		0	8.02 × 10^9^	~0	1.91 × 10^9^
		**5″-OH**							
		**2-NH**							
**Position**			**RAF (H_4_A)**						
			**1**				**2**		
			$∆G_{a}^{RAF}$		$k_{ZCT\_0}^{RAF}$		$∆G_{a}^{RAF}$	$k_{ZCT\_0}^{RAF}$	
**C-3**			38		4.26 × 10^7^		48	7.98 × 10^5^	
**C-5**			51		2.95 × 10^5^		56	4.30 × 10^4^	
**C-6**			38		4.20 × 10^7^		45	2.74 × 10^6^	
**C-7**			60		3.32 × 10^3^		63	2.34 × 10^3^	
**C-8**			39		3.12 × 10^7^		41	1.13 × 10^7^	
**C-9**			50		3.54 × 10^5^		53	1.11 × 10^5^	
**C-10**			43		5.28 × 10^6^		47	1.23 × 10^6^	
**C-1′**			42		9.00 × 10^6^		45	2.24 × 10^6^	
**C-1″**			37		6.37 × 10^7^		35	1.20 × 10^8^	
**C-2″**			39		3.21 × 10^7^		29	5.69 × 10^8^	
**C-3″**			22		1.67 × 10^8^		47	1.09 × 10^6^	
**C-4″**			38		3.89 × 10^7^		29	7.41 × 10^7^	
**C-5″**			38		3.97 × 10^7^		39	2.45 × 10^7^	
**C-6″**			28		7.29 × 10^7^		25	1.39 × 10^7^	

## Data Availability

Data is contained within the article and Supplementary Material.

## Supplementary Materials

The following supporting information can be downloaded at: https://www.mdpi.com/article/10.3390/antiox13020143/s1, Figure S1: Deprotonation route, estimated pKa values and molar fractions of acid-base species of **1** at physiological pH (7.4); Figure S2: Deprotonation route, estimated pKa values and molar fractions of acid-base species of **2** at physiological pH (7.4); Figure S3: Spin density distribution maps (0.002 electrons/bohr^3^) of formed radical species formed in the reaction between **H_4_A** of compounds **1** (a,c) and **2** (b,d) and HO^•^ radical. Blue color represents positive spin density values; Figure S4: Optimized geometries of radical adducts with characteristic intramolecular distances (Å) formed between **H_4_A** of compound **1** and HO^•^; Figure S5: Optimized geometries of radical adducts with characteristic intramolecular distances (Å) formed between **H_4_A** of compound **2** and HO^•^; Figure S6: HOMO (*Highest Occupied Molecular Orbital*) orbitals of formed phenoxide anions formed in the reaction between **H_4_A** of compounds **1** (a,c) and **2** (b,d) and HO^−^; Figure S7: HOMO orbitals for the corresponding **H_2_A^•−^** nad **H_2_A^2•−^** species formed in the reaction of proton transfer from **H_3_A^•^** (top) and **H_3_A^•−^** (middle) of compound **1** to HO^−^ as well as from **H_3_A^•^** of compound **2** (bottom) to HO^−^; Figure S8: Optimized geometries of neutral **H_2_A** products of compounds **1** (top) and **2** (middle) and monoanionic **HA^−^** product (bottom) formed in the reaction between **H_3_A^•^** and **H_2_A^•−^** and HO^•^; Figure S9: Optimized geometries of adducts with characteristic intramolecular distances (Å) formed between **H_3_A^•^** of compound **1** and HO^•^; Figure S10: Optimized geometries of adducts with characteristic intramolecular distances (Å) formed between **H_3_A^•^** of compound **2** and HO^•^; Figure S11: Optimized geometries of adducts with characteristic intramolecular distances (Å) formed between **H_3_A^•−^** of compound **1** and HO^•^; Figure S12: Optimized transition state geometries for HAT/PCET reaction pathways between neutral **H_4_A** of compound **1** (top) and **2** (bottom) and HO^•^ with characteristic intraatomic distances (Å); Figure S13: Dependence of the total energy (a.u.) on the HO–H2 characteristic distance (Å) for the HAT/PCET mechanism between neutral **H_4_A** of compound **1** and HO**^•^**; Figure S14: Optimized transition state geometries for the RAF mechanism between neutral **H_4_A** of compound **1** and HO^•^ at the M06-2X/6-311++G(d,p) level of theory with characteristic intermolecular distances (Å); Figure S15: Optimized transition state geometries for the RAF mechanism between neutral **H_4_A** of compound **2** and HO^•^ at the M06-2X/6-311++G(d,p) level of theory with characteristic intermolecular distances (Å); Figure S16: Graph of the dependence of ln*k*_TST_ (green line) and ln*k*_ZCT_0_ (blue line) on the reciprocal values of temperature for the RAF mechanism (**1** (top) and **2** (bottom)); Figure S17: Dependence of the total energy (a.u.) on the HO–H4 characteristic distance (Å) for the SPL mechanism between neutral **H_4_A** of compound **1** (left) and **2** (right) and HO^−^; Figure S18: Energy profile for the HAT/PCET reaction pathway between **H_3_A^•^** of compound **2** and HO^•^ (left) as well as **H_2_A^•−^** of compound **1** and HO^•^ (right) in the singlet (blue) and triplet (red) spin states. Interatomic distances are given in angstroms [Å]; Figure S19: Energy profile for the RCF reaction path between **H_3_A^•^** of compound **1** and HO^•^ in the singlet (blue) and triplet (red) spin states. Interatomic distances are given in angstroms [Å]; Table S1: 2D geometries of the investigated acid-base species of **1** compound that participate in the various investigated mechanisms of antiradical action. The black color describes the thermodynamically favored products obtained between neutral species **H_4_A** and HO^•^. The red color describes the favored products obtained between monoanionic species **H_3_A^−^**(**H_3_A^•^**) and HO^•^, while the blue color describes favored products obtained between dianionic species **H_2_A^2−^**(**H_2_A^•−^**) and HO^•^; Table S2: 2D geometries of the investigated acid-base species of compound **2** that participate in the various investigated mechanisms of antiradical action. The black color describes the thermodynamically favored products obtained between neutral species **H_4_A** and HO^•^. The red color describes the favored products obtained between monoanionic species **H_3_A^−^**(**H_3_A^•^**) and HO^•^; Table S3: Estimated values of kinetic parameters: activation energy (Δ*G*_a_, kJ mol^−1^), rate constants of the bimolecular chemical reactions (M^−1^s^−1^) between the investigated compounds and HO^•^ estimated by the Transition State Theory (*k*_TST_); Table S4: Estimation values of overall rate constants (*k*_overall_), relative antiradical capacity (*r*^T^) and branching ratios, Γ_i_ (%), for exergonic reaction pathways evaluated at physiological conditions, pH = 7.4.
