# Supplementary material for: Mechanism of Antiradical Activity of Coumarin-Trihydroxybenzohydrazide Derivatives: A Comprehensive Kinetic DFT Study
Source: Antioxidants (Basel). 2024 Jan 24;13(2):143. doi: 10.3390/antiox13020143 (PMC10885972; doi:10.3390/antiox13020143)
Supplement: Supplementary file 1 [file antioxidants-13-00143-s001.zip › antioxidants-2797819-supplementary.pdf]

Supplementary Material

for

Mechanism of antiradical activity of coumarin-trihydroxybenzohydrazide derivatives: A comprehensive kinetic DFT study

Žiko Milanović [1], Dušan Dimić [2], Edina H. Avdović [1], Dušica M. Simijonović [1], Đura Nakarada [2], Vladimir Jakovljević [3], and Radiša Vojinović [3,*], Zoran S. Marković [1,4]

[1]*University of Kragujevac, Institute for Information Technologies, Department of Science, Liceja Kneževine Srbije 1A, 34000 Kragujevac, Serbia;*

[2]*University of Belgrade, Faculty of Physical Chemistry, Studentski trg 12-16, 11000 Belgrade, Serbia;*

[3]*University of Kragujevac, Faculty of Medical Sciences, Svetozara Markovića 69, 34000 Kragujevc, Republic of Serbia;*

[4]*State University of Novi Pazar, Department of Natural Science and Mathematics, Vuka Karadžića bb, 36300, Novi Pazar, Serbia, Serbia;*

**Correspondence: rhvojinovic@medf.kg.ac.rs; Tel.: +381 34 306 800*

To quantify the molar fractions of acid-base species, the relationship between the acid constants (*Ka*) and the *p*Ka values as well as the expression for the equilibrium constant of the deprotonation process was used:

$$Ka_1 = 10^{-pKa1} \tag{1s}$$

$$Ka_2 = 10^{-pKa2} \tag{2s}$$

$$Ka_3 = 10^{-pKa3} \tag{3s}$$

$$Ka_4 = 10^{-pKa4} \tag{4s}$$

$$Ka_1 = \frac{\left[H_3A^{-}\right]\left[H^{+}\right]}{\left[H_4A\right]} \tag{5s}$$

$$Ka_2 = \frac{\left[H_2A^{2-}\right]\left[H^{+}\right]}{\left[H_3A^{-}\right]} \tag{6s}$$

$$Ka_3 = \frac{\left[HA^{3-}\right]\left[H^{+}\right]}{\left[H_2A^{2-}\right]} \tag{7s}$$

$$Ka_4 = \frac{\left[A^{4-}\right]\left[H^{+}\right]}{\left[HA^{3-}\right]} \tag{8s}$$

Molar fractions (*f*) of the represented acid-base species, were calculated using the following equations:

$$f(A^{4-}) = \frac{1}{1+\beta_1\left[H^{+}\right]+\beta_2\left[H^{+}\right]^2+\beta_2\left[H^{+}\right]^2+\beta_4\left[H^{+}\right]^4} \tag{9s}$$

$$f(HA^{3-}) = \beta_1\left[H^+\right]f(A^{4-}) \tag{10s}$$

$$f(H_2A^{2-}) = \beta_2\left[H^+\right]^2 f(HA^{3-}) \tag{11s}$$

$$f(H_3A^{-}) = \beta_3\left[H^+\right]^3 f(H_2A^{2-}) \tag{12s}$$

where [H+] represents the concentration of hydrogen ions at physiological pH in this case ($[H^+]=3.98\times10^{-8}$ M), while β represents global formation equilibrium constants:

$$\beta_1 = 10^{pKa4} \tag{13s}$$

$$\beta_2 = 10^{pKa4+pKa3} \tag{14s}$$

$$\beta_3 = 10^{pKa4+pKa3+pKa2} \tag{15s}$$

$$\beta_4 = 10^{pKa4+pKa3+pKa2+pKa1} \tag{16s}$$

The overall rate constants (k_{overall}) for the reactions between the investigated compounds and $HO^{\bullet}$ were calculated by the following equations:

$$k_{overall} = f(H_4A)\times k_{TOT}^{H_4A} + f(H_3A^{-})\times k_{TOT}^{H_3A^{-}} + f(H_2A^{2-})\times k_{TOT}^{H_2A^{2-}} + f(HA^{3-})\times k_{TOT}^{HA^{3-}} + f(A^{4-})\times k_{TOT}^{A^{4-}} \tag{17s}$$

Due to the very small proportion of acid-base species at physiological pH, eq. 17s can be represented as:

$$k_{overall}^{Comp1} = f(H_4A) \times k_{TOT}^{H_4A} + f(H_3A^-) \times k_{TOT}^{H_3A^-} + f(H_2A^{2-}) \times k_{TOT}^{H_2A^{2-}} \quad (18s)$$

$$k_{overall}^{Comp2} = f(H_4A) \times k_{TOT}^{H_4A} + f(H_3A^-) \times k_{TOT}^{H_3A^-} \quad (19s)$$

where k_{TOT} represent the sum of the favorable reaction pathways for the investigation species and $HO^{\bullet}$:

$$k_{TOT} = k_{HAT} + k_{RAF} + k_{SPL} + k_{ET} \quad (20s)$$

Antioxidative capacity r^T of the examined antioxidant relative to trolox (**Tx**)/gallic acid (**GA**) ($k_{overall}^{Ref}$) was calculated using the formula:

$$r^T = \frac{k_{overall}}{k_{overall}^{\mathrm{Re}f}} \quad (21s)$$

The relative amounts of products (%), i.e. branching ratios (Γi) were determined:

$$\Gamma_i = \frac{k_i}{k_{overall}} \quad (22s)$$

to evaluate which of the mechanistic pathways, i, is dominant.

Standard mechanisms of antioxidant action between neutral forms of the investigated compounds and $HO^{\bullet}$ radical:

HAT/PCET: $H_4A + HO^{\bullet} \rightarrow H_3A^{\bullet} + H_2O$ (23s)

RAF: $H_4A + HO^{\bullet} \rightarrow [H_4A\text{-}OH]^{\bullet}$ (24s)

SET: $H_4A + HO^{\bullet} \rightarrow H_4A^{\bullet+} + HO^{-}$ (25s)

PT: $H_4A^{\bullet+} + HO^{-} \rightarrow H_3A^{\bullet} + H_2O$ (26s)

SPL: $H_4A + HO^{-} \rightarrow H_3A^{-} + H_2O$ (27s)

ET: $H_3A^{-} + HO^{\bullet} \rightarrow H_3A^{\bullet} + HO^{-}$ (28s)

where H_4A, $H_3A^{\bullet}$, $[HO\text{-} H_4A]^{\bullet}$, $H_4A^{\bullet+}$, and H_3A^{-} denote neutral species, its radical, radical adduct, radical cation, and anion.

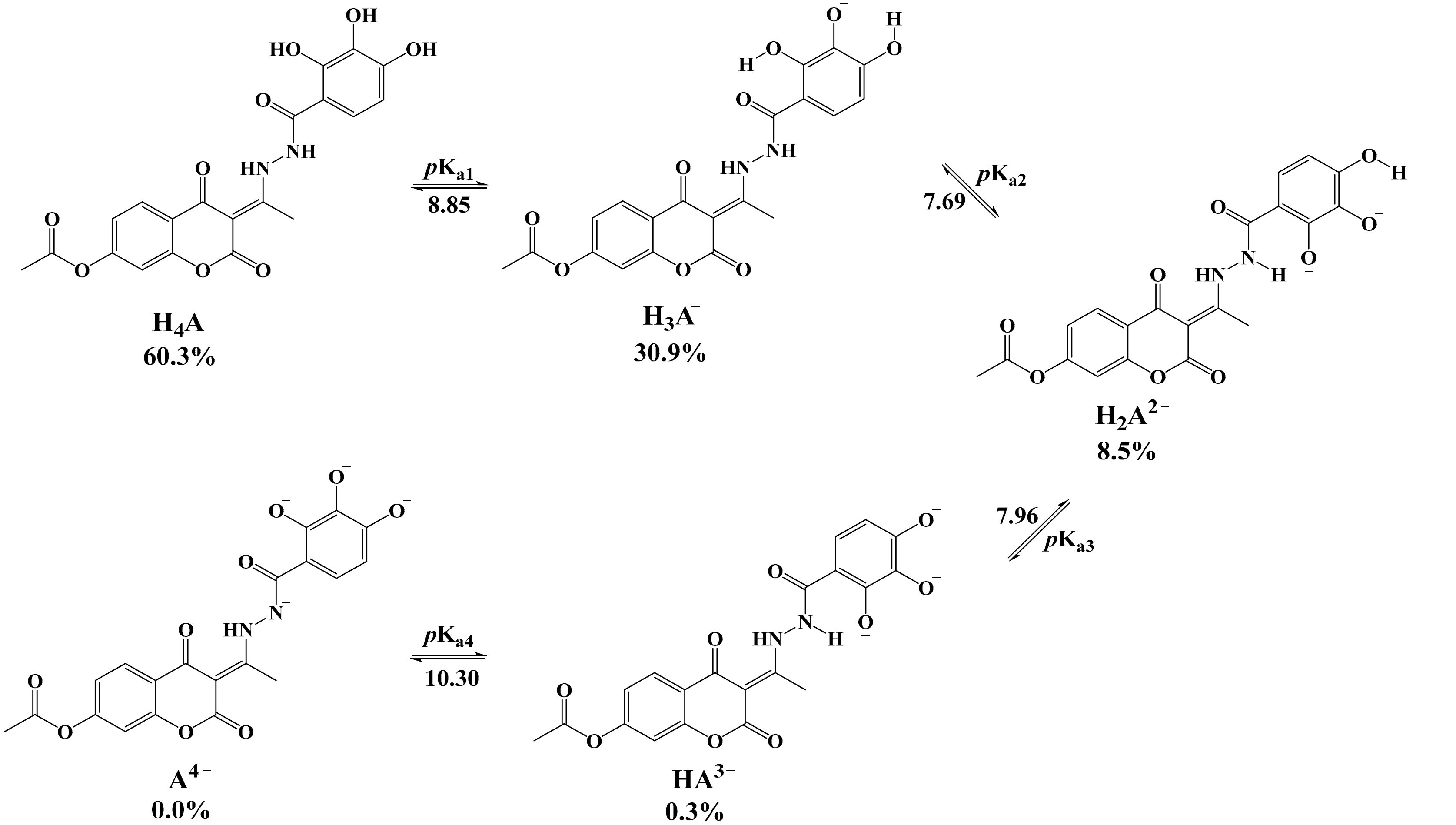

Figure S1. Deprotonation route, estimated *p*K*a* values and molar fractions of acid-base species of **1** at physiological pH (7.4)

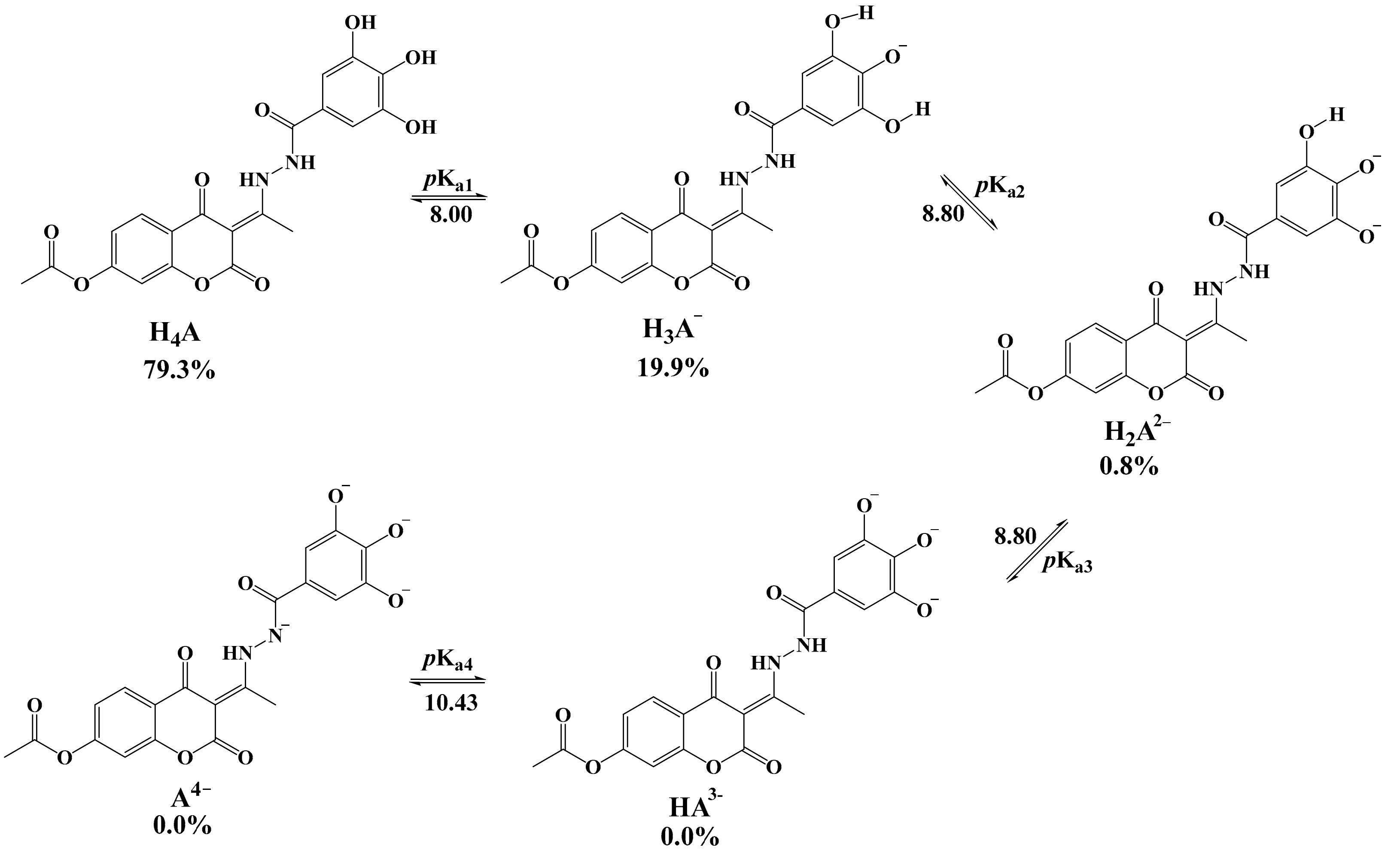

Figure S2. Deprotonation route, estimated pKa values and molar fractions of acid-base species of **2** at physiological pH (7.4)

Table S1. 2D geometries of the investigated acid-base species of **1** compound that participate in the various investigated mechanisms of antiradical action. The black color describes the thermodynamically favored products obtained between neutral species **H_4A** and $HO^{\bullet}$. The red color describes the favored products obtained between monoanionic species **H_3A^-**(**$H_3A^{\bullet}$**) and $HO^{\bullet}$, while the blue color describes favored products obtained between dianionic species **H_2A^{2-}**(**$H_2A^{\bullet-}$**) and $HO^{\bullet}$.

<table>
<tr><td colspan="4">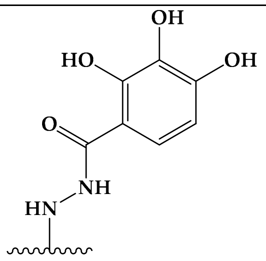
H₄A</td></tr>
<tr><td colspan="4">SPLET (H₄A)</td></tr>
<tr><td>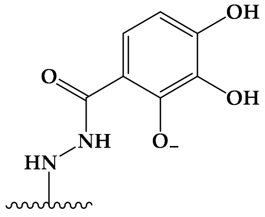</td><td>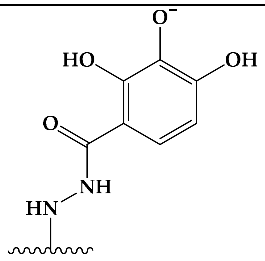</td><td>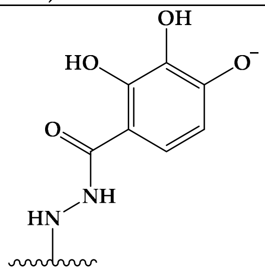</td><td>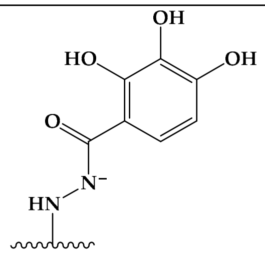</td></tr>
<tr><td>2″-OH</td><td>3″-OH</td><td>4″-OH</td><td>N2-H</td></tr>
<tr><td colspan="4">HAT/PCET (H₄A)</td></tr>
<tr><td>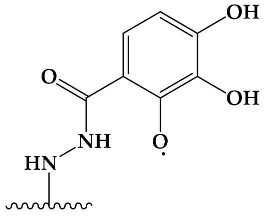</td><td>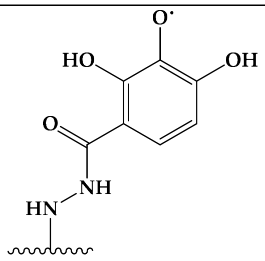</td><td>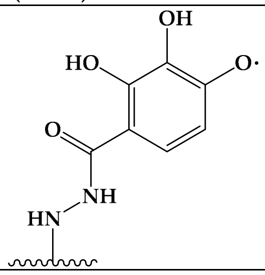</td><td>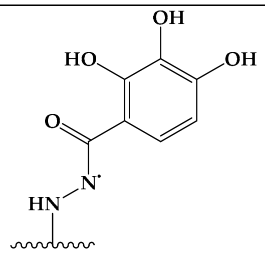</td></tr>
<tr><td>2″-OH</td><td>3″-OH</td><td>4″-OH</td><td>N2-H</td></tr>
<tr><td colspan="4">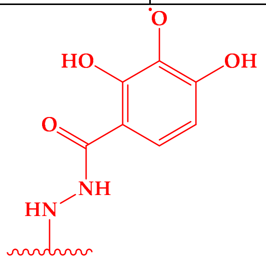</td></tr>
</table>

<table>
<tr><td colspan="6">$H_3A^{\bullet}$/ H_3A^{-}</td></tr>
<tr><td colspan="6">HAT/PCET ($H_3A^{\bullet}$/ H_3A^{-})</td></tr>
<tr><td colspan="2">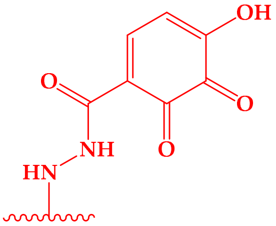</td><td colspan="2">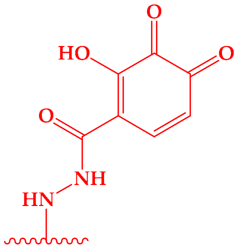</td><td colspan="2">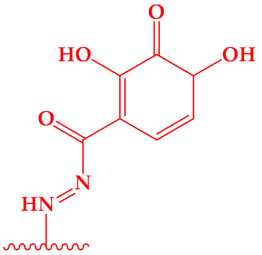</td></tr>
<tr><td colspan="2">2″-OH</td><td colspan="2">4″-OH</td><td colspan="2">N2-H</td></tr>
<tr><td colspan="6">PT ($H_3A^{\bullet}$/ H_3A^{-})</td></tr>
<tr><td colspan="2">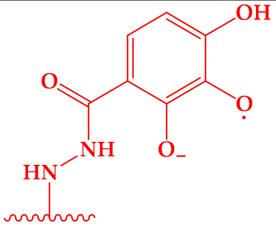</td><td colspan="2">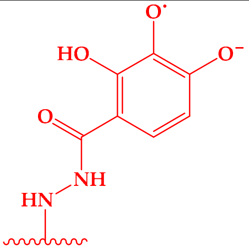</td><td colspan="2">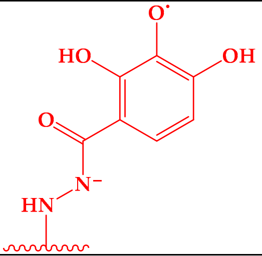</td></tr>
<tr><td colspan="2">2″-OH</td><td colspan="2">4″-OH</td><td colspan="2">N2-H</td></tr>
<tr><td colspan="6">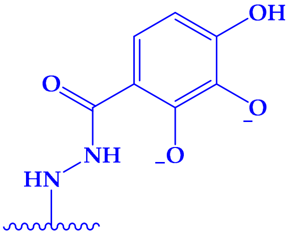
H_2A^{2-}/ $H_2A^{\bullet -}$</td></tr>
<tr><td colspan="6">HAT/PCET (H_2A^{2-}/ $H_2A^{\bullet -}$)</td></tr>
<tr><td colspan="3">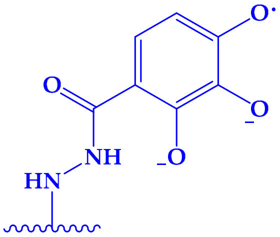</td><td colspan="3">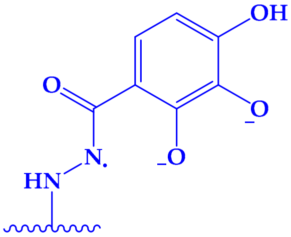</td></tr>
<tr><td colspan="3">4″-OH</td><td colspan="3">2-NH/2″-OH</td></tr>
<tr><td colspan="6">PT (H_2A^{2-}/ $H_2A^{\bullet -}$)</td></tr>
</table>

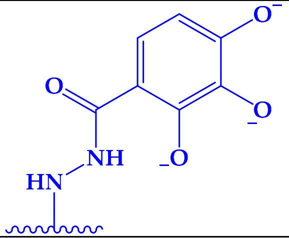	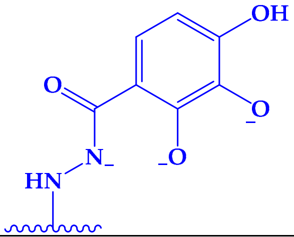

Table S2. 2D geometries of the investigated acid-base species of compound **2** that participate in the various investigated mechanisms of antiradical action. The black color describes the thermodynamically favored products obtained between neutral species **H_4A** and $HO^•$. The red color describes the favored products obtained between monoanionic species **H_3A^-**(**$H_3A^•$**) and $HO^•$.

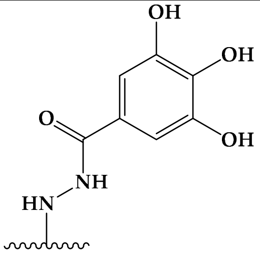 H_4A		
SPLET (H_4A)		
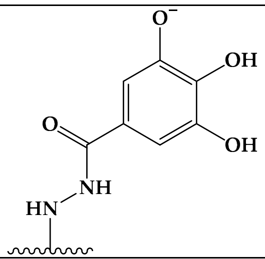	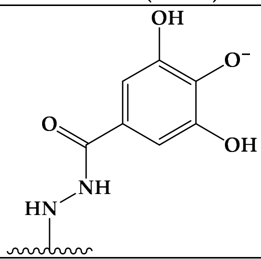	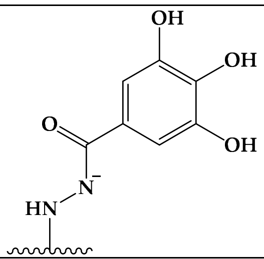
3″-OH/5″-OH	4″-OH	N2-H
HAT/PCET (H_4A)		
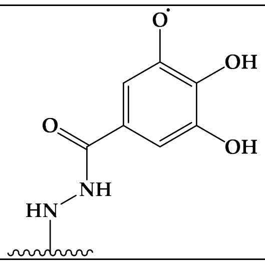	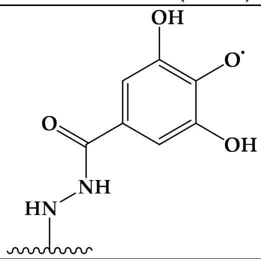	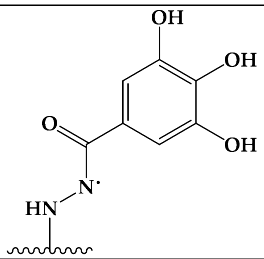

<table>
<tr><td colspan="3">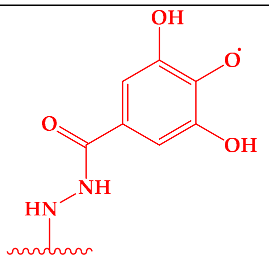
H_3A^-</td></tr>
<tr><td colspan="3">HAT/PCET ($H_3A^\bullet$/ H_3A^-)</td></tr>
<tr><td>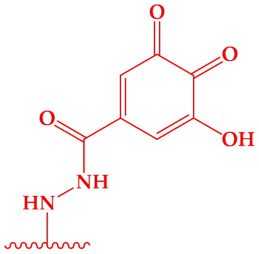</td><td>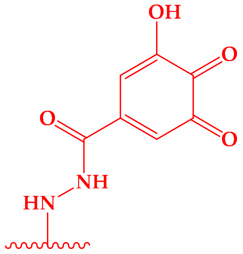</td><td>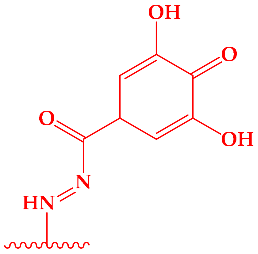</td></tr>
<tr><td>3″-OH</td><td>5″-OH</td><td>N2-H</td></tr>
<tr><td colspan="3">PL ($H_3A^\bullet$/ H_3A^-)</td></tr>
<tr><td>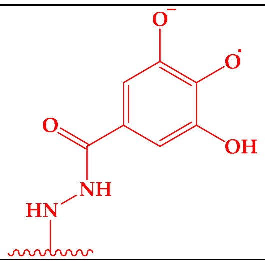</td><td>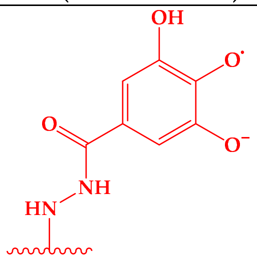</td><td>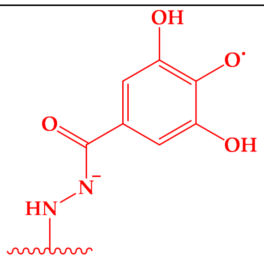</td></tr>
<tr><td>3″-OH</td><td>5″-OH</td><td>N2-H</td></tr>
</table>

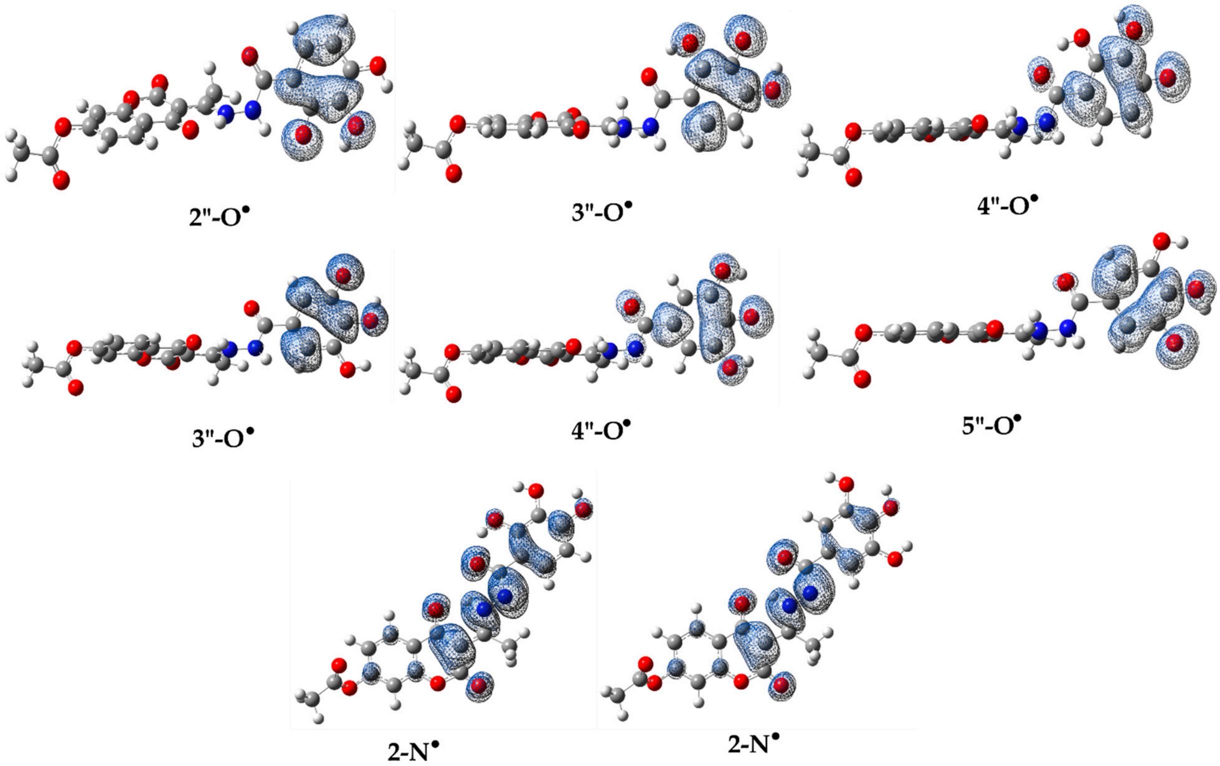

Figure S3. Spin density distribution maps (0.002 electrons/bohr^3) of formed radical species formed in the reaction between **H$_4$A** of compounds **1** (a,c) and **2** (b,d) and HO$^•$ radical. Blue color represents positive spin density values.

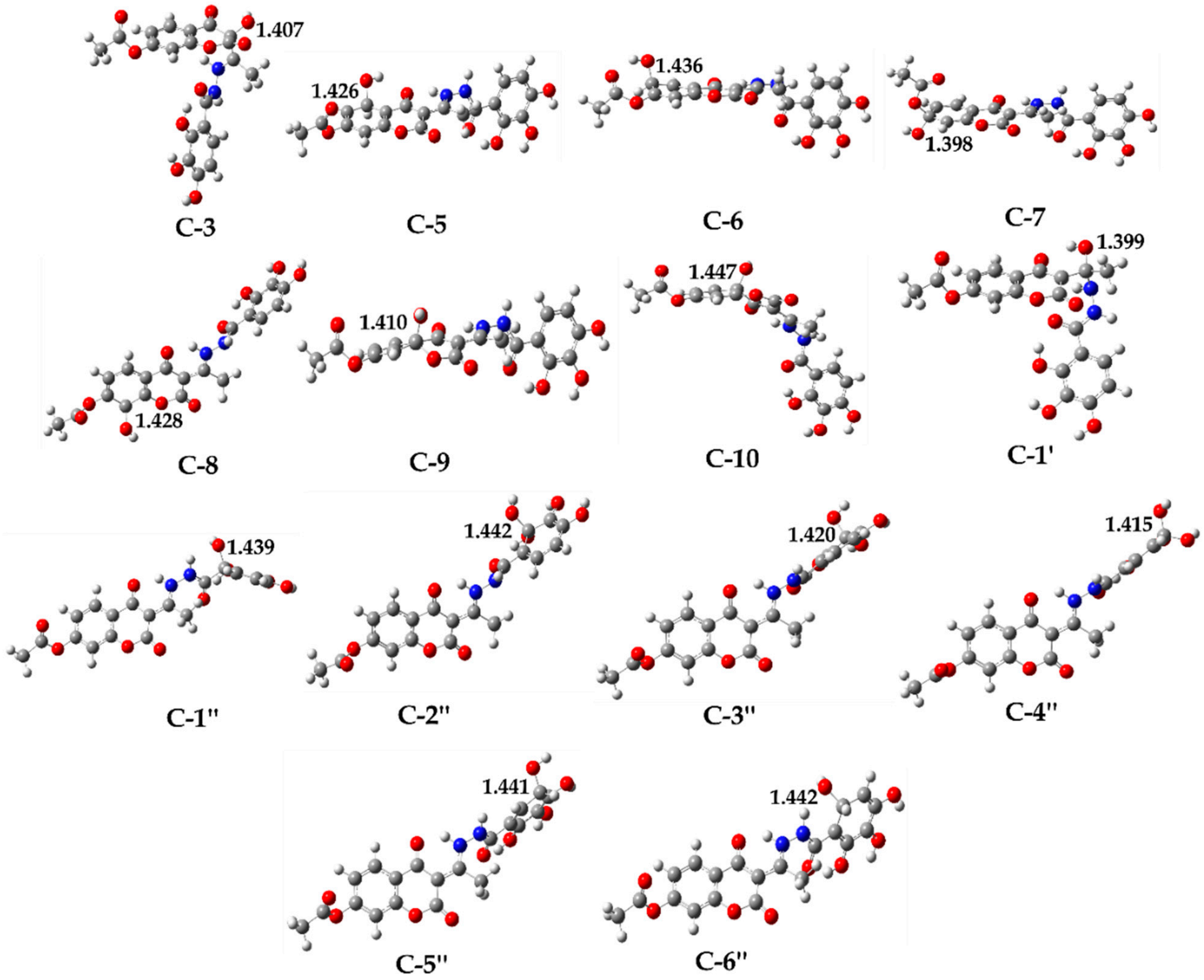

Figure S4. Optimized geometries of radical adducts with characteristic intramolecular distances (Å) formed between **H4A** of compound **1** and HO•

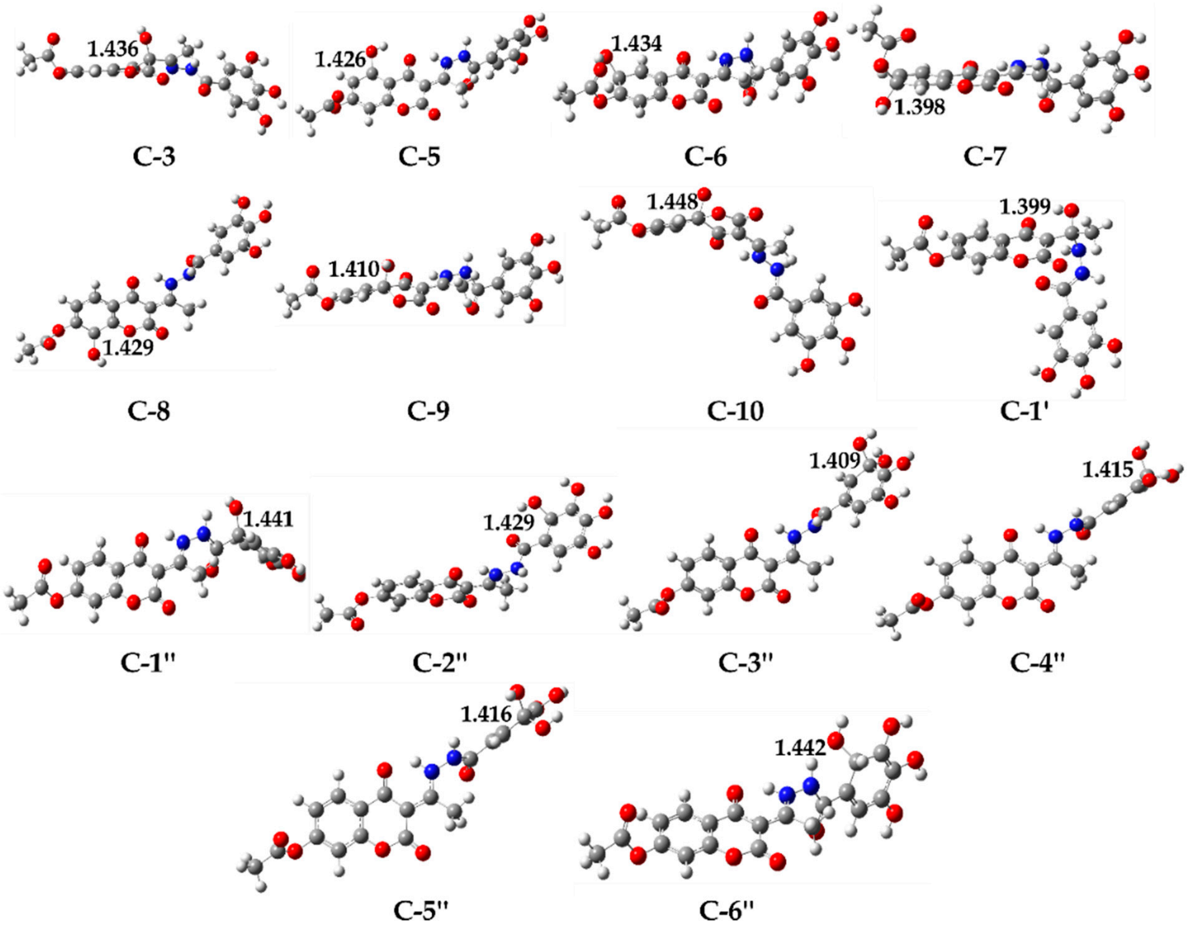

Figure S5. Optimized geometries of radical adducts with characteristic intramolecular distances (Å) formed between **H$_4$A** of compound **2** and HO$^•$

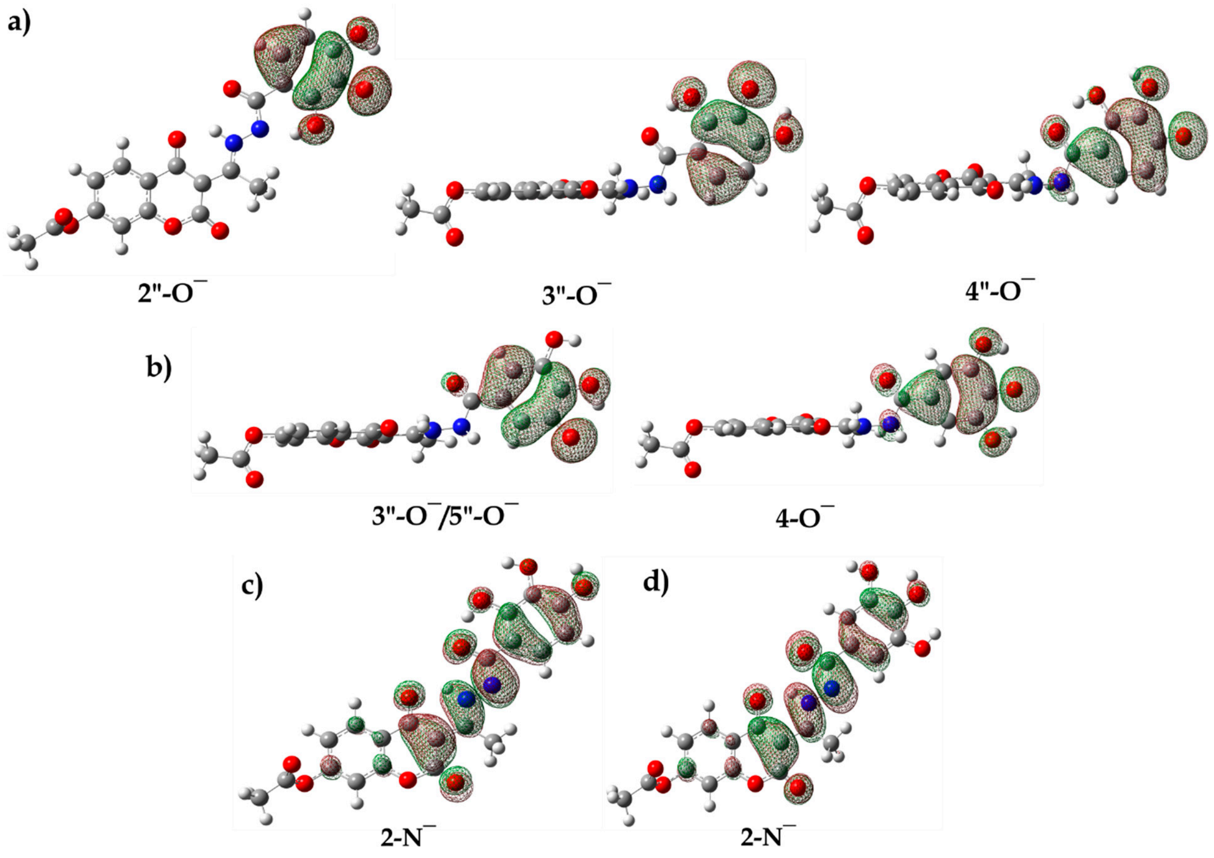

Figure S6. HOMO (*Highest Occupied Molecular Orbital*) orbitals of formed phenoxide anions formed in the reaction between **H_4A** of compounds **1** (a,c) and **2** (b,d) and HO^-

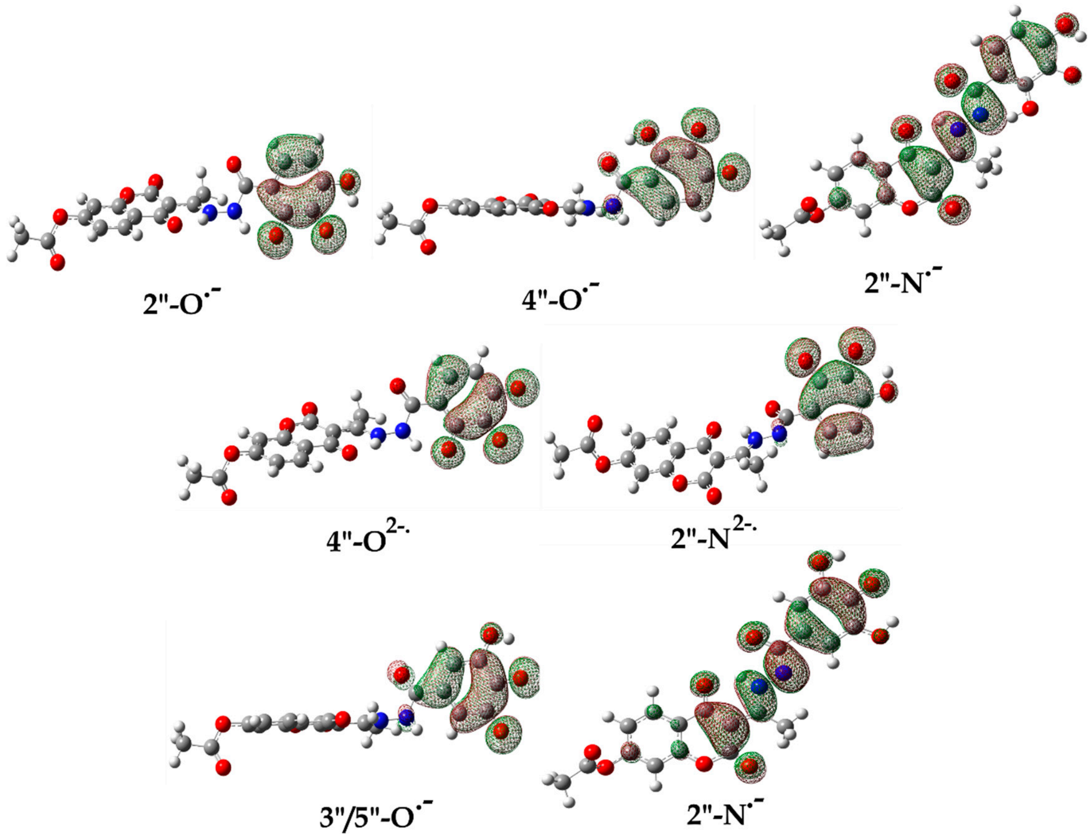

Figure S7. HOMO orbitals for the corresponding $\mathbf{H_2A^{\bullet -}}$ nad $\mathbf{H_2A^{2\bullet -}}$ species formed in the reaction of proton transfer from $\mathbf{H_3A^{\bullet}}$ (top) and $\mathbf{H_3A^{\bullet -}}$ (middle) of compound **1** to HO^- as well as from $\mathbf{H_3A^{\bullet}}$ of compound **2** (bottom) to HO^-.

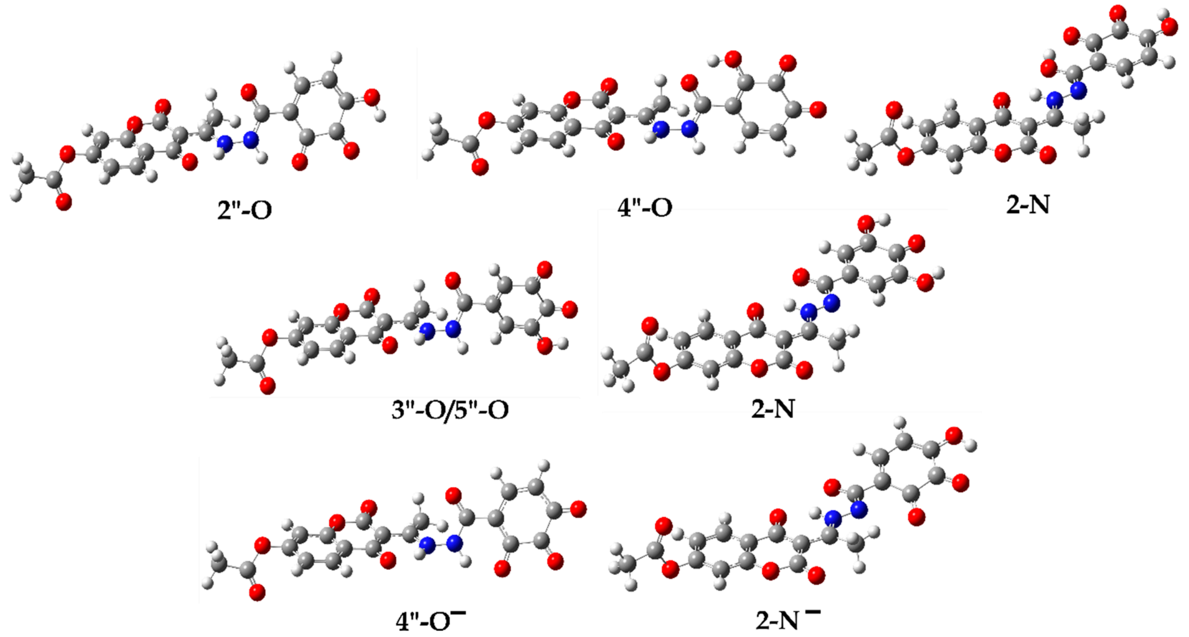

Figure S8. Optimized geometries of neutral $\mathbf{H_2A}$ products of compounds **1** (top) and **2** (middle) and monoanionic $\mathbf{HA^-}$ product (bottom) formed in the reaction between $\mathbf{H_3A^\bullet}$ and $\mathbf{H_2A^{\bullet-}}$ and $HO^\bullet$.

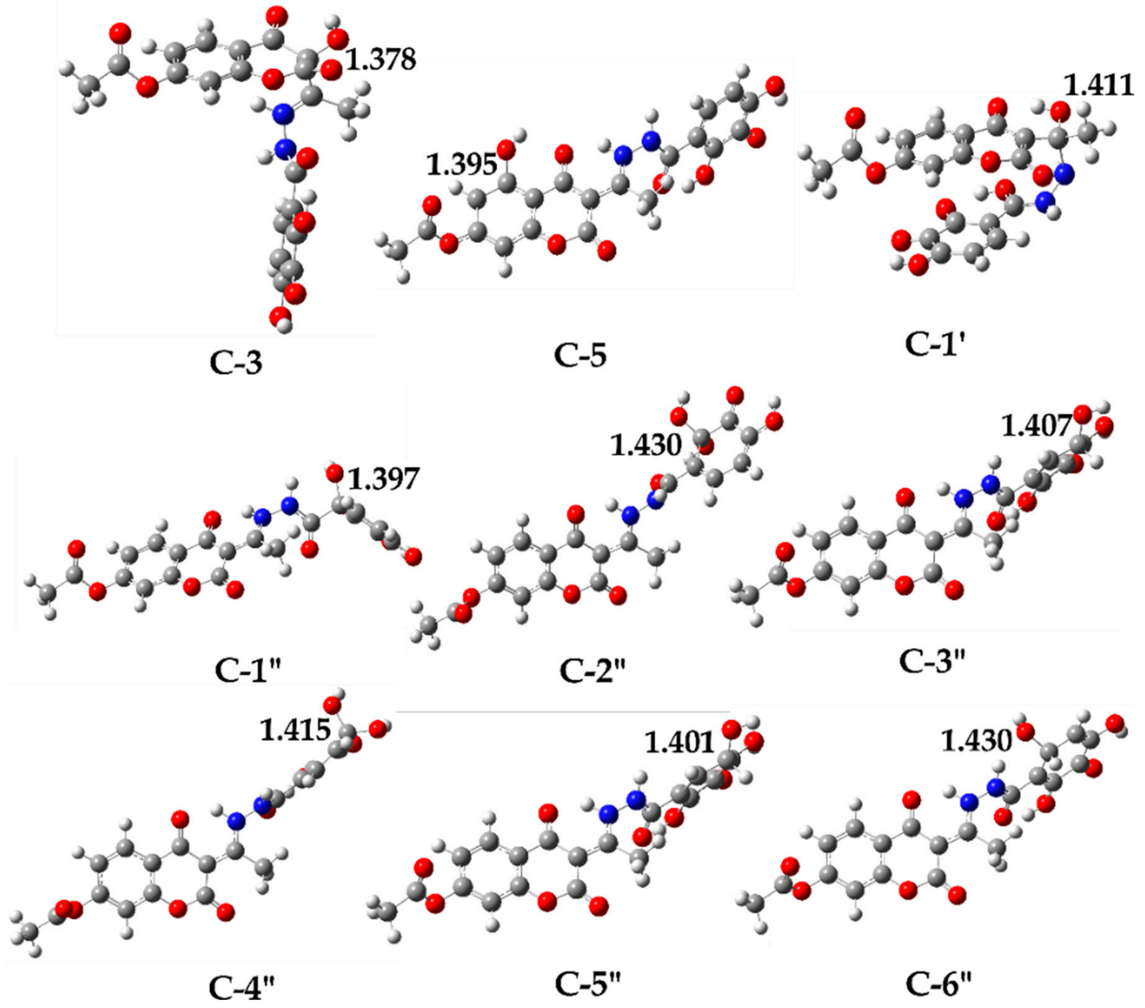

Figure S9. Optimized geometries of adducts with characteristic intramolecular distances (Å) formed between **$H_3A^•$** of compound **1** and $HO^•$

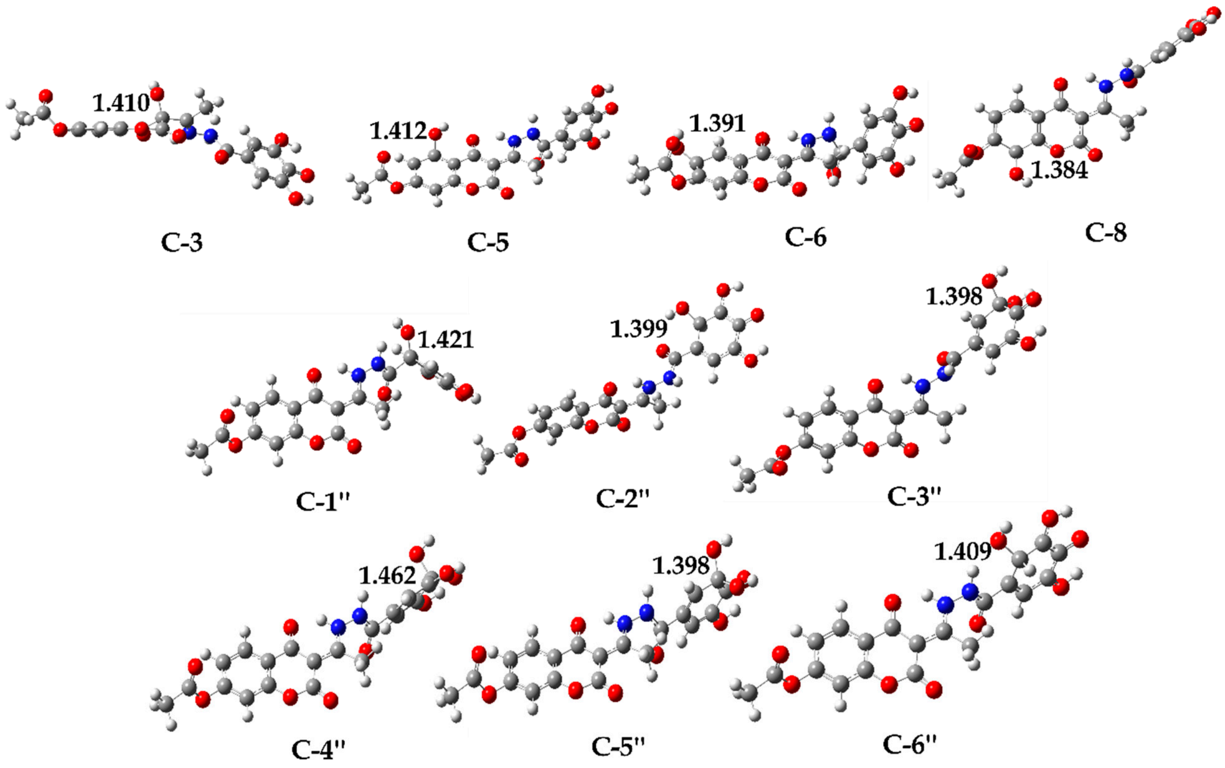

Figure S10. Optimized geometries of adducts with characteristic intramolecular distances (Å) formed between **H$_3$A$^•$** of compound **2** and HO$^•$

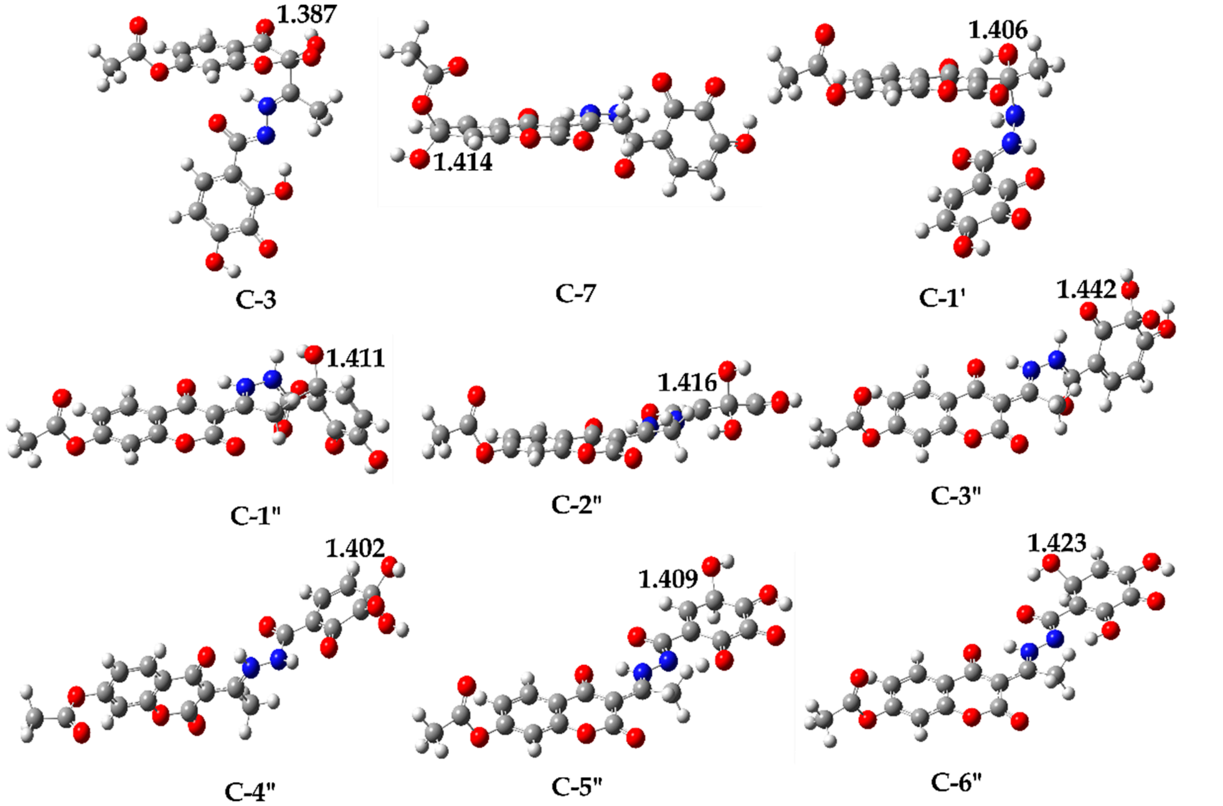

Figure S11. Optimized geometries of adducts with characteristic intramolecular distances (Å) formed between $\mathbf{H_3A^{\bullet -}}$ of compound **1** and $HO^{\bullet}$

Table S3. Estimated values of kinetic parameters: activation energy (ΔG_a, kJ mol^{-1}), rate constants of the bimolecular chemical reactions (M^{-1}s^{-1}) between the investigated compounds and HO$^{\bullet}$ estimated by the Transition State Theory (k_{TST}).

**HAT/PCET**				
**1/2** Position	**1 (H_4A)**		**2 (H_4A)**	
	ΔG_a^{HAT}	k_{TST}^{HAT}	ΔG_a^{HAT}	k_{TST}^{HAT}
**2″-OH/3″-OH**	58	9.53×10^{3}	59	6.79×10^{3}
**3″-OH/5″-OH**	41	8.26×10^{6}		
**4″-OH/4″-OH**	55	6.28×10^{4}	54	6.28×10^{4}
Position	**RAF**			
	**1 (H_4A)**		**2 (H_4A)**	
	ΔG_a^{RAF}	k_{TST}^{RAF}	ΔG_a^{RAF}	k_{TST}^{RAF}
**C-3**	38	3.29×10^{7}	48	6.19×10^{5}
**C-5**	51	1.88×10^{5}	56	2.72×10^{4}
**C-6**	38	3.26×10^{7}	45	2.13×10^{6}
**C-7**	60	1.60×10^{3}	63	1.53×10^{3}
**C-8**	39	2.59×10^{7}	41	9.33×10^{6}
**C-9**	50	2.27×10^{5}	53	7.10×10^{4}
**C-10**	43	4.01×10^{6}	47	9.34×10^{5}
**C-1′**	42	7.23×10^{6}	45	1.80×10^{6}
**C-1"**	37	5.60×10^{7}	35	1.02×10^{8}
**C-2"**	39	2.71×10^{7}	29	1.40×10^{9}
**C-3"**	22	1.91×10^{10}	47	9.00×10^{5}
**C-4"**	38	3.17×10^{7}	29	1.53×10^{9}
**C-5"**	38	3.38×10^{7}	39	2.08×10^{7}
**C-6"**	28	2.06×10^{7}	25	6.80×10^{9}

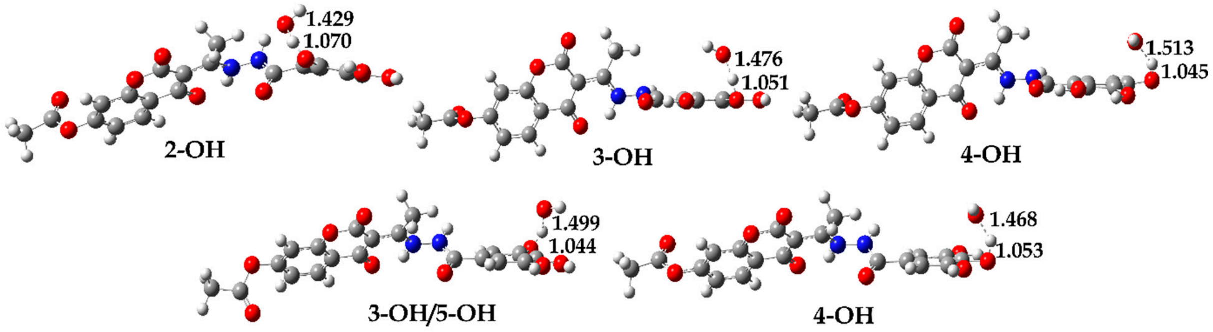

Figure S12. Optimized transition state geometries for HAT/PCET reaction pathways between neutral **H₄A** of compound **1** (top) and **2** (bottom) and HO$^{\bullet}$ with characteristic intraatomic distances (Å)

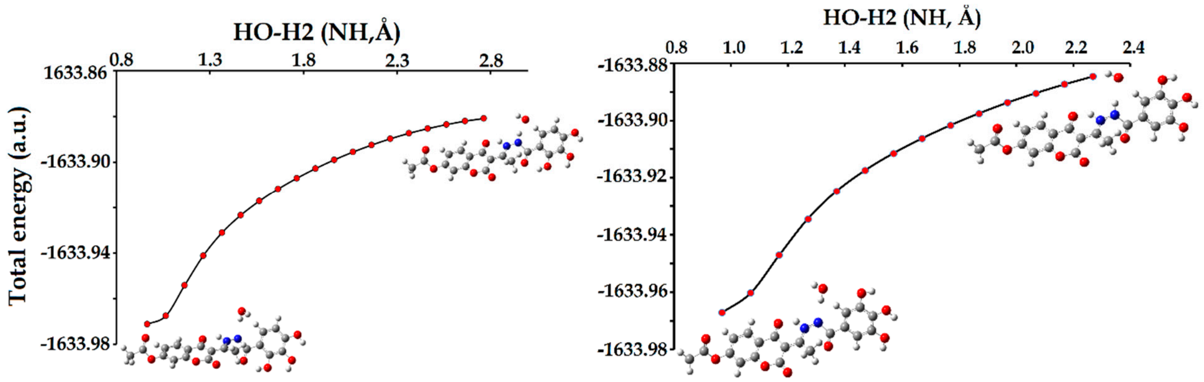

Figure S13. Dependence of the total energy (a.u.) on the HO–H2 characteristic distance (Å) for the HAT/PCET mechanism between neutral **H₄A** of compound **1** (left) and **2** (right) and HO$^{\bullet}$

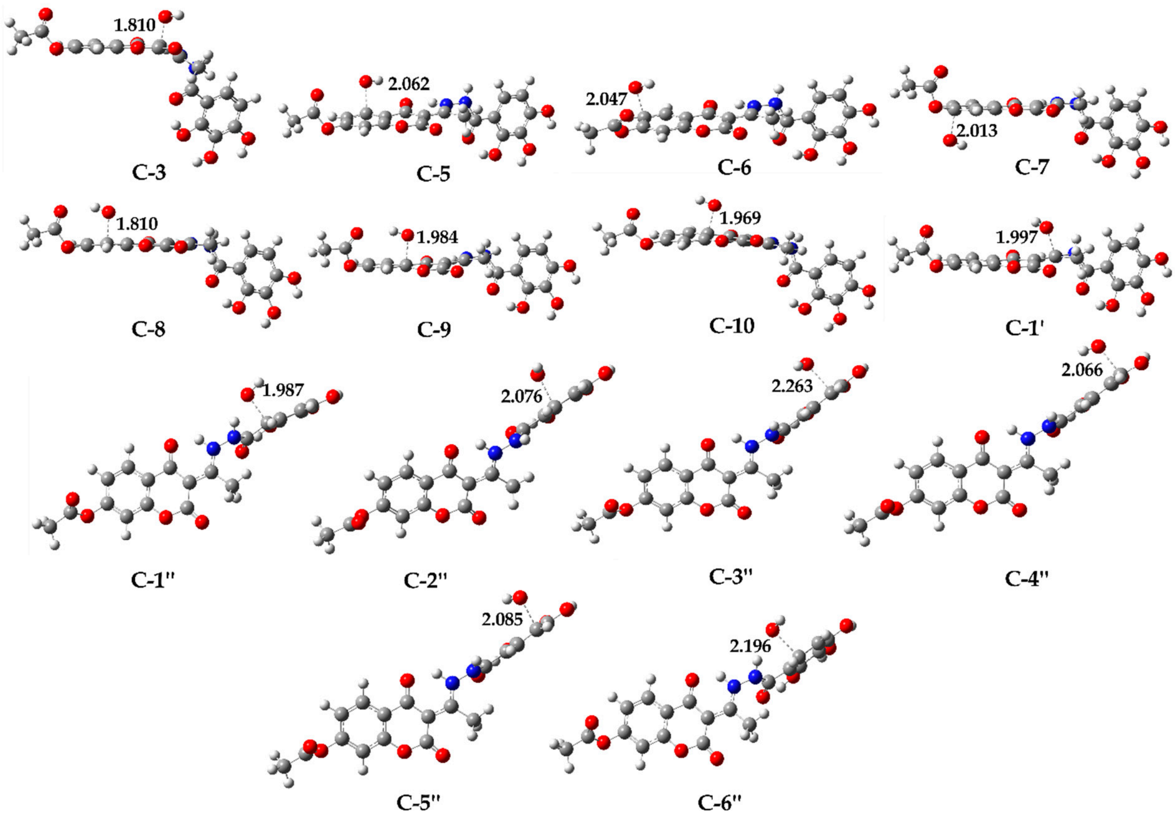

Figure S14. Optimized transition state geometries for the RAF mechanism between neutral **H_4A** of compound **1** and $HO^{\bullet}$ at the M06-2X/6-311++G(d,p) level of theory with characteristic intermolecular distances (Å)

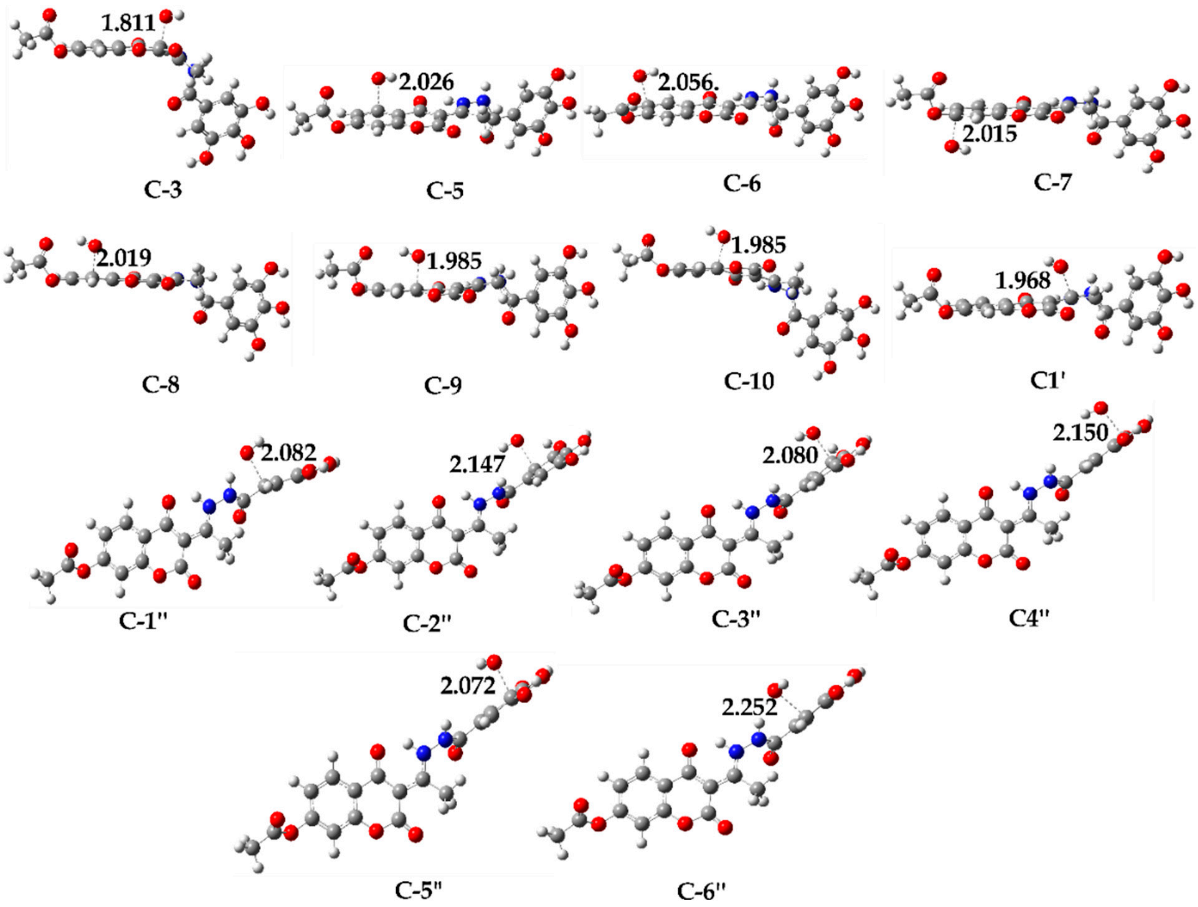

Figure S15. Optimized transition state geometries for the RAF mechanism between neutral **H₄A** of compound **2** and HO• at the M06-2X/6-311++G(d,p) level of theory with characteristic intermolecular distances (Å)

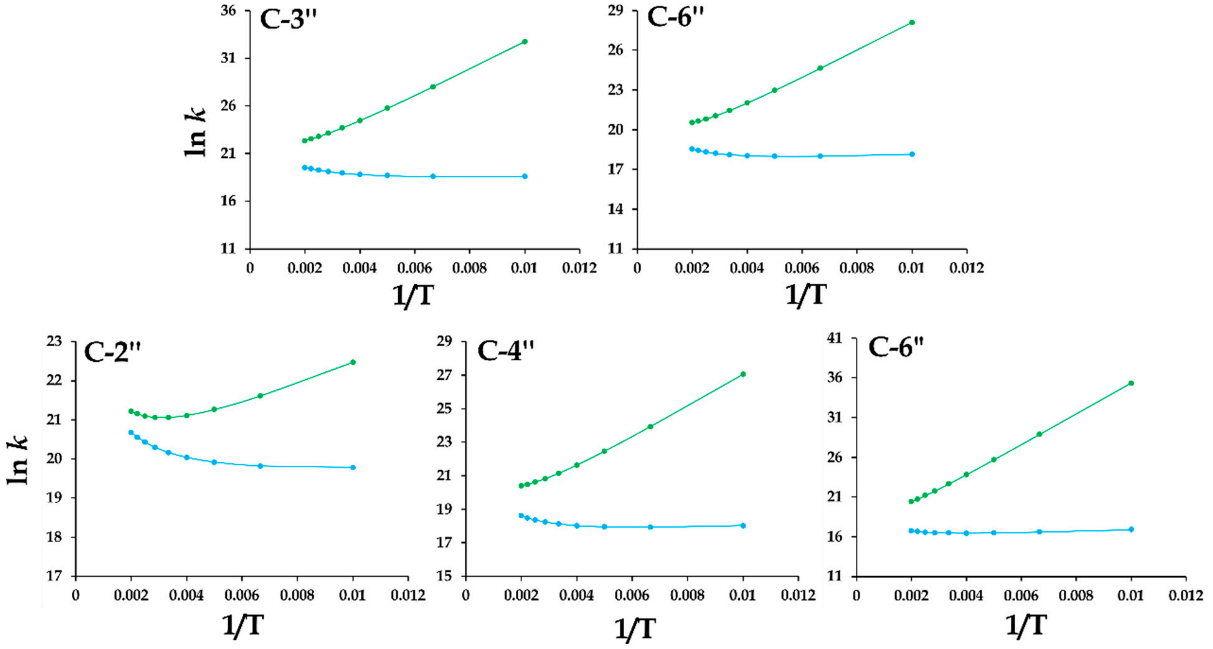

Figure S16. Graph of the dependence of lnk_{TST} (green line) and lnk_{ZCT_0} (blue line) on the reciprocal values of temperature for the RAF mechanism (**1** (top) and **2** (bottom))

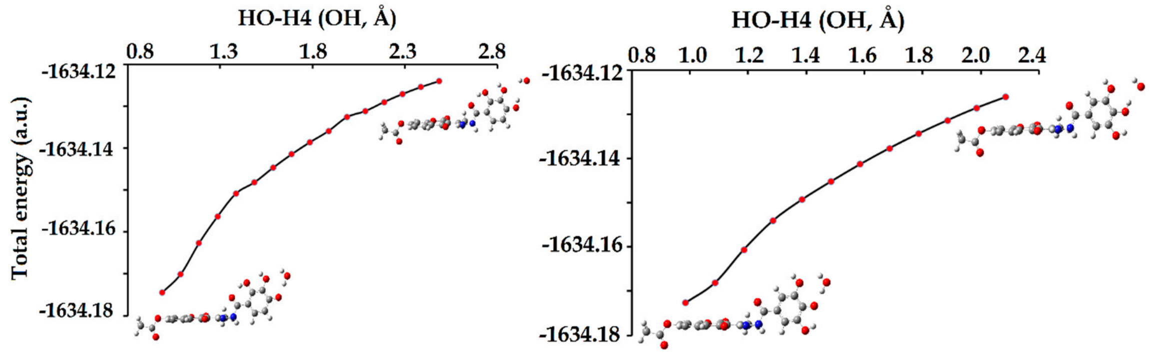

Figure S17. Dependence of the total energy (a.u.) on the HO–H4 characteristic distance (Å) for the SPL mechanism between neutral **H_4A** of compound **1** (left) and **2** (right) and HO^-

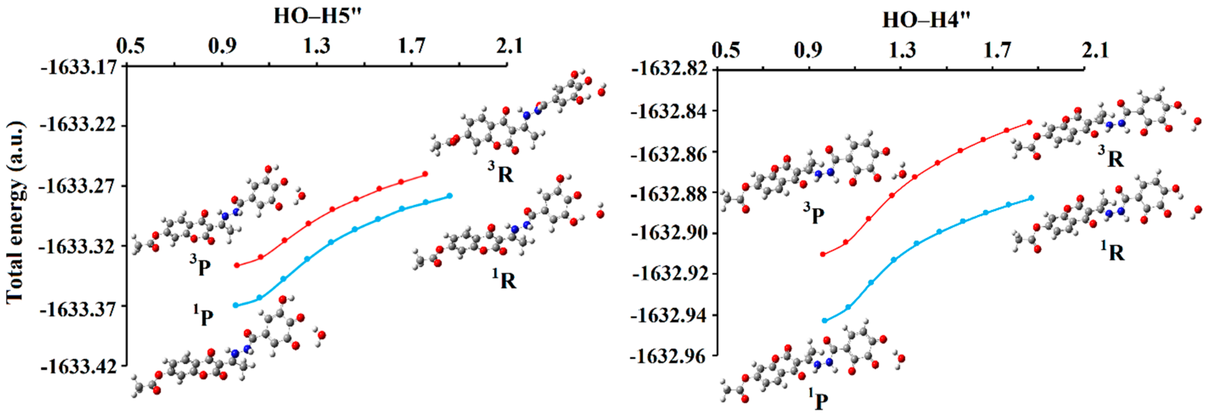

Figure S18. Energy profile for the HAT/PCET reaction pathway between $\mathbf{H_3A^{\bullet}}$ of compound **2** and $HO^{\bullet}$ (left) as well as $\mathbf{H_2A^{\bullet -}}$ of compound **1** and $HO^{\bullet}$ (right) in the singlet (blue) and triplet (red) spin states. Interatomic distances are given in angstroms [Å]

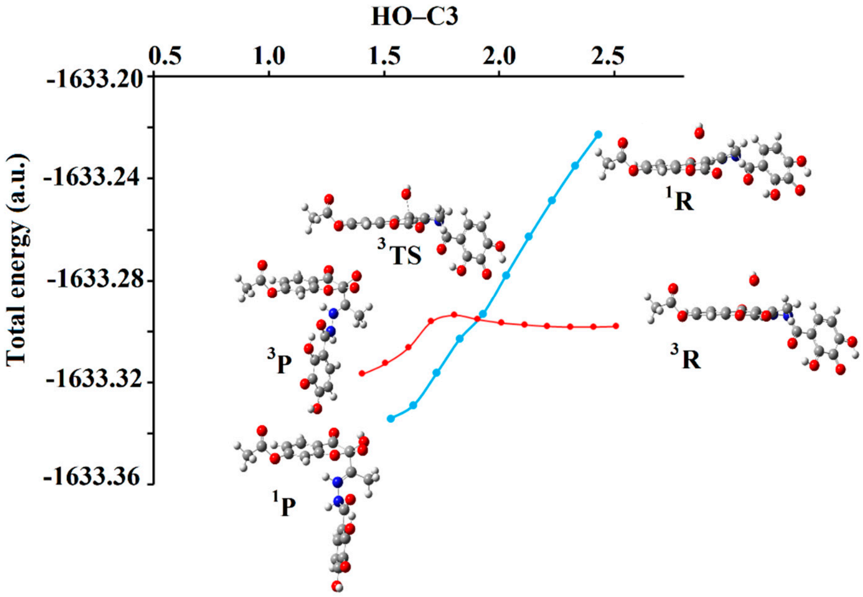

Figure S19. Energy profile for the RCF reaction path between $\mathbf{H_3A^{\bullet}}$ of compound **1** and $HO^{\bullet}$ in the singlet (blue) and triplet (red) spin states. Interatomic distances are given in angstroms [Å]

Table S4. Estimation values of overall rate constants ($k_{overall}$), relative antiradical capacity (r^T) and branching ratios, Γ_i (%), for exergonic reaction pathways evaluated at physiological conditions, pH=7.4.

<table>
<tr><td rowspan="2">Mechanism</td><td rowspan="2">Position</td><td colspan="3">Compound **1**</td><td colspan="2">Compound **2**</td></tr>
<tr><td>**H_4A**</td><td>**H_3A^-**</td><td>**H_2A^{2-}**</td><td>**H_4A**</td><td>**H_3A^-**</td></tr>
<tr><td rowspan="4">**HAT/PCET**</td><td>**2″-OH/3″-OH**</td><td>0.0</td><td>1.6</td><td>/</td><td>0.0</td><td>0.9</td></tr>
<tr><td>**3″-OH/4″-OH**</td><td>0.0</td><td>/</td><td>/</td><td>0.0</td><td>/</td></tr>
<tr><td>**4″-OH/5″-OH**</td><td>0.0</td><td>1.6</td><td>0.4</td><td>0.0</td><td>0.9</td></tr>
<tr><td>**2-NH**</td><td>3.1</td><td>1.6</td><td>0.4</td><td>3.7</td><td>0.9</td></tr>
<tr><td rowspan="14">**RAF/RCF**</td><td>**C-3**</td><td>0.1</td><td rowspan="14">22.0</td><td rowspan="14">5.6</td><td>0.0</td><td rowspan="14">12.6</td></tr>
<tr><td>**C-5**</td><td>0.0</td><td>0.0</td></tr>
<tr><td>**C-6**</td><td>0.1</td><td>0.0</td></tr>
<tr><td>**C-7**</td><td>0.0</td><td>0.0</td></tr>
<tr><td>**C-8**</td><td>0.1</td><td>0.0</td></tr>
<tr><td>**C-9**</td><td>0.0</td><td>0.0</td></tr>
<tr><td>**C-10**</td><td>0.0</td><td>0.0</td></tr>
<tr><td>**C-1′**</td><td>0.0</td><td>0.0</td></tr>
<tr><td>**C-1"**</td><td>0.1</td><td>0.2</td></tr>
<tr><td>**C-2"**</td><td>0.1</td><td>1.1</td></tr>
<tr><td>**C-3"**</td><td>0.3</td><td>0.0</td></tr>
<tr><td>**C-4"**</td><td>0.1</td><td>0.1</td></tr>
<tr><td>**C-5"**</td><td>0.1</td><td>0.0</td></tr>
<tr><td>**C-6"**</td><td>0.1</td><td>0.0</td></tr>
<tr><td>**SPL/SET**</td><td>***n*-OH/*n*-NH**</td><td>12.3</td><td>6.2</td><td>0.4</td><td>14.7</td><td>3.9</td></tr>
<tr><td rowspan="4">**ET/PT**</td><td>**2″-O/3″-O**</td><td>12.9</td><td>1.6</td><td rowspan="2">/</td><td>15.5</td><td>0.9</td></tr>
<tr><td>**3″-O/4″-O**</td><td>12.2</td><td>/</td><td>15.5</td><td>/</td></tr>
<tr><td>**4″-O/5″-O**</td><td>12.9</td><td>1.6</td><td rowspan="2">0.4</td><td>15.3</td><td>0.9</td></tr>
<tr><td>**2-N**</td><td>0.2</td><td>1.6</td><td>11.3</td><td>0.9</td></tr>
<tr><td colspan="2">$k_{overall}$</td><td colspan="3">**3.75×10^{10}**</td><td colspan="2">**4.11×10^{10}**</td></tr>
<tr><td colspan="2">r^T_{Tx}</td><td colspan="3">**19.1**</td><td colspan="2">**21.2**</td></tr>
<tr><td colspan="2">r^T_{GA}</td><td colspan="3">**1.5**</td><td colspan="2">**1.6**</td></tr>
</table>
